# Fundamental Roles of the Golgi-Associated *Toxoplasma* Aspartyl Protease, ASP5, at the Host-Parasite Interface

**DOI:** 10.1371/journal.ppat.1005211

**Published:** 2015-10-16

**Authors:** Pierre-Mehdi Hammoudi, Damien Jacot, Christina Mueller, Manlio Di Cristina, Sunil Kumar Dogga, Jean-Baptiste Marq, Julia Romano, Nicolò Tosetti, Juan Dubrot, Yalin Emre, Matteo Lunghi, Isabelle Coppens, Masahiro Yamamoto, Daniel Sojka, Paco Pino, Dominique Soldati-Favre

**Affiliations:** 1 Department of Microbiology and Molecular Medicine, Centre Médical Universitaire, University of Geneva, Geneva, Switzerland; 2 Department of Immunoparasitology, Research Institute for Microbial Diseases, Osaka University, Osaka, Japan; 3 Department of Chemistry, Biology and Biotechnology, University of Perugia, Perugia, Italy; 4 Department of Molecular Microbiology and Immunology, Johns Hopkins University Bloomberg School of Public Health, Baltimore, Maryland, United States of America; 5 Department of Pathology and Immunology, Centre Médical Universitaire, University of Geneva, Geneva, Switzerland; 6 Institute of Parasitology, Biology Centre of the Academy of Sciences of the Czech Republic, České Budějovice, Czech Republic; University of Wisconsin Medical School, UNITED STATES

## Abstract

*Toxoplasma gondii* possesses sets of dense granule proteins (GRAs) that either assemble at, or cross the parasitophorous vacuole membrane (PVM) and exhibit motifs resembling the HT/PEXEL previously identified in a repertoire of exported *Plasmodium* proteins. Within *Plasmodium spp*., cleavage of the HT/PEXEL motif by the endoplasmic reticulum-resident protease Plasmepsin V precedes trafficking to and export across the PVM of proteins involved in pathogenicity and host cell remodelling. Here, we have functionally characterized the *T*. *gondii* aspartyl protease 5 (ASP5), a Golgi-resident protease that is phylogenetically related to Plasmepsin V. We show that deletion of *ASP5* causes a significant loss in parasite fitness *in vitro* and an altered virulence *in vivo*. Furthermore, we reveal that ASP5 is necessary for the cleavage of GRA16, GRA19 and GRA20 at the PEXEL-like motif. In the absence of ASP5, the intravacuolar nanotubular network disappears and several GRAs fail to localize to the PVM, while GRA16 and GRA24, both known to be targeted to the host cell nucleus, are retained within the vacuolar space. Additionally, hypermigration of dendritic cells and bradyzoite cyst wall formation are impaired, critically impacting on parasite dissemination and persistence. Overall, the absence of ASP5 dramatically compromises the parasite’s ability to modulate host signalling pathways and immune responses.

## Author Summary

The opportunistic pathogen *Toxoplasma gondii* infects a large range of nucleated cells where it replicates intracellularly within a parasitophorous vacuole (PV) surrounded by a membrane (PVM). Parasites constitutively secrete dense-granule proteins (GRAs) both into and beyond the PV which participate in remodelling of the PVM, recruitment of host organelles, neutralization of the host cellular defences, and subversion of host cell functioning. In addition, the GRAs critically contribute to cyst wall formation, a process that critically ensures parasite persistence and transmission. To act as effector molecules, some of the GRAs must be translocated across the PVM. Within the related apicomplexan parasite *P*. *falciparum*, a repertoire of proteins exported beyond the PVM contain a motif cleaved by a specific protease, Plasmepsin V. Examination of the repertoire of GRAs in *T*. *gondii* revealed that some proteins exhibit such export-like motifs suggestive of protease involvement. In this study, we have functionally characterized the related aspartyl protease 5 (TgASP5) in both virulent and persistent *T*. *gondii* strains, and have investigated the phenotypic consequences of its deletion in the context of overall parasite biology, its intracellular niche, the infected host cells and the murine model. Our findings revealed fundamental roles of TgASP5 at the host-parasite interface.

## Introduction

The phylum Apicomplexa groups obligate protozoan parasites that are the causative agents of severe diseases in humans and animals such as malaria, toxoplasmosis, babesiosis and coccidiosis. The key process of invasion and subsequent multiplication within their host cells is maintained via secretion from three distinct phylum-specific organelles termed the micronemes, rhoptries and dense granules [1–3].

*Plasmodium falciparum* is the most notorious member of the Apicomplexa in terms of its impact upon human health [4]. During its intraerythrocytic stage development, *P*. *falciparum* modulates the infected red blood cell by exporting a large repertoire of proteins to impact notably on nutrient acquisition, rosetting and cytoadherence [5]. This host cell modulation is governed by the export of various effector proteins, many of which contain a plasmodium export element RxLxE/Q/D (PEXEL), whilst a smaller repertoire of exported proteins lack this motif and are thus termed PEXEL-negative exported proteins [6]. The protease underpinning this cleavage event, Plasmepsin V, is an integral membrane protein localized to the endoplasmic reticulum (ER) in an orientation such that the catalytic aspartyl protease domain faces the ER lumen [7]. Plasmepsin V cleaves the PEXEL motif after the leucine residue, ensuring secretion of the effectors into the host erythrocyte and subsequent parasite survival [8–10].

*Toxoplasma gondii* is amongst the most widely distributed parasites with nearly half of the human population chronically infected. Infection during pregnancy can result in severe neurological birth defects, whilst fatal cerebral toxoplasmosis can occur in association with immunosuppressive diseases and treatments. *T*. *gondii* follows a complex life cycle involving a haploid replicative stage, followed by chronic encystation in a broad range of intermediate hosts, and meiosis in the intestine of the definitive felid host [11]. Intermediate hosts are infected either by ingestion of either oocysts shed by felids, or by bradyzoites in tissue cysts within infected meat. The fast-replicating tachyzoites are responsible for the acute stage of infection and dissemination into all tissues, whereas the slow growing bradyzoite stage establishes chronic infection with resultant cysts predominantly found in the brain and striated muscle.

Following host cell invasion, *T*. *gondii* tachyzoites and bradyzoites are surrounded by a PVM that resists endo/lysosomal fusion [12] and secludes them from the host cell cytosol. Unlike rhoptry effector proteins that are secreted at the onset of invasion, dense granule proteins (GRAs) are secreted once the parasite resides within the PV. GRAs have been implicated in a variety of processes linked to the establishment of parasitism, including the formation of the membranous nanotubular network (MNN) produced within the PV at the posterior side of invading parasites [13, 14]. More recently, several GRAs have been demonstrated to cross the PVM and subvert host cellular functions. Specifically, GRA15 modulates host cell signalling pathways by NF-kB nuclear translocation and NF-kB–mediated transcription of cytokines and other effector molecules [15]. GRA16 reaches the host cell nucleus, where it positively modulates genes involved in cell cycle progression and the p53 tumour suppressor pathway [16]. GRA24 modulates the early immune response to infection by promoting host p38 MAPK activation [17]. The activity of these proteins within the host cell is suggestive of the presence of an export pathway similar to that of *Plasmodium spp*. In this context, the *T*. *gondii* genome was reported to contain genes exhibiting a signal peptide in combination with proximal sequences reminiscent of the PEXEL motif (PEXEL-like motif) [18]. Some of these genes encode novel dense granule proteins; GRA19, GRA20 and GRA21, that are cleaved at the PEXEL-like motif, however do not cross the PVM and are instead incorporated into the PV and PVM. Furthermore, the previously reported GRA3, GRA5, and GRA15 were also found to contain the exact PEXEL consensus motif RxLxD/E, whilst most of the other identified GRAs possess an N-terminal motif resembling the pattern RxLxD/E within approximately 140 residues of their predicted start methionine [18].

The presence of PEXEL-like motifs on several GRAs spoke for the existence of an aspartyl protease involved in cleavage and export of these effector proteins in *T*. *gondii*. Of the seven aspartic protease paralogues encoded by the *T*. *gondii* genome (ASPs), ASP5 and ASP7 are members of the evolutionarily distinguished group (D) of apicomplexan aspartic proteases comprising Plasmepsin V [19]. Since ASP7 is not expressed in tachyzoites and bradyzoites, ASP5 emerges as the most promising candidate to cleave *T*. *gondii* PEXEL-like motif containing proteins. Here we report the functional characterization of *ASP5* in *T*. *gondii* type I and type II strains. We demonstrate that ASP5 is responsible for the cleavage of GRAs containing PEXEL-like motifs and is necessary for the export of GRAs beyond the PVM into the host cells. We also report the broader phenotypic consequences of the absence of ASP5: severely compromised parasite fitness, a block in the formation of the MNN, an inability to enhance dendritic cell (DC) migration, a dramatic remodelling of the host immune response, and an impairment in cyst wall formation without any impact on tachyzoite to bradyzoite conversion.

## Results

### ASP5 is a Golgi-resident aspartyl protease that contributes to parasite fitness

**Fig 1:**
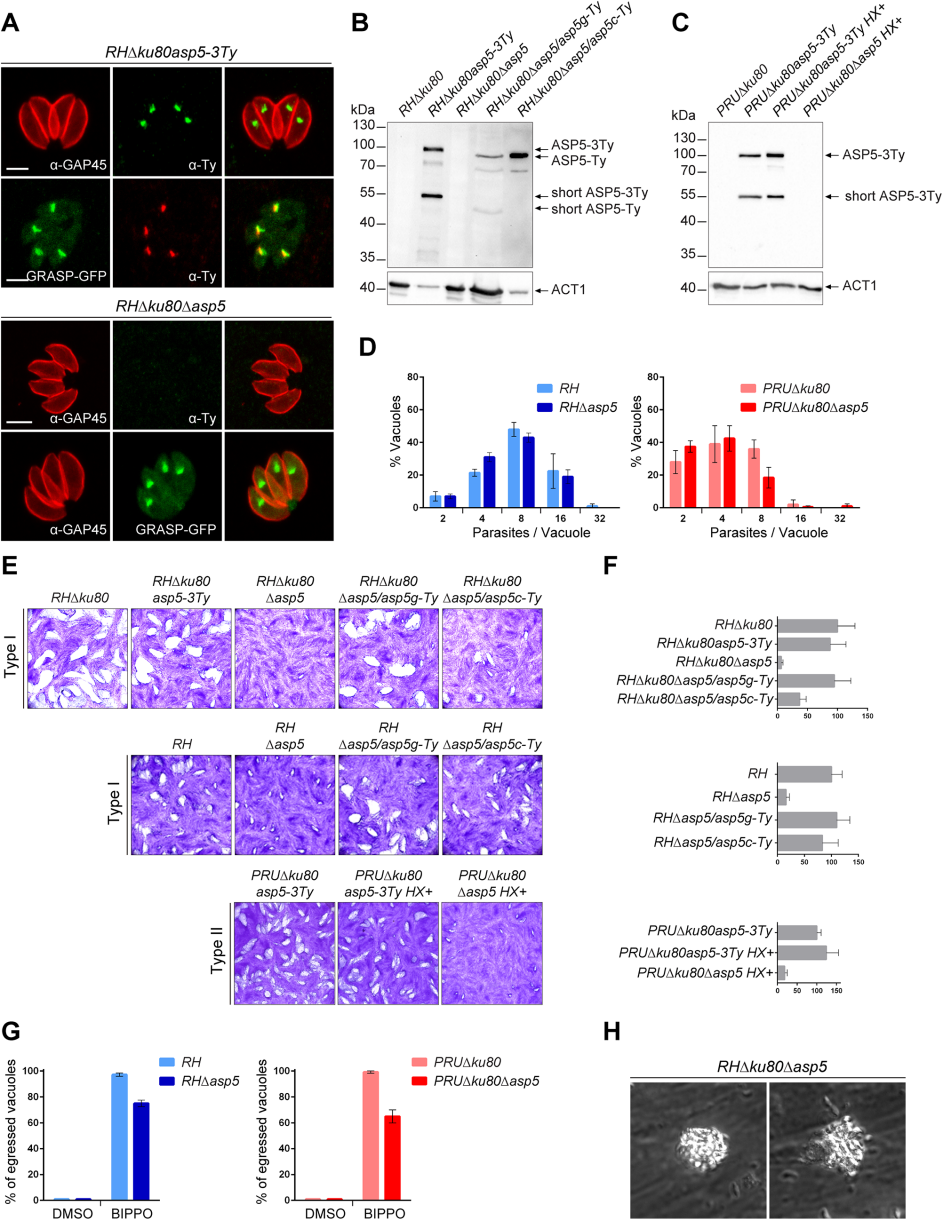
ASP5 is a dispensable Golgi-resident protein. (A) In type I parasites, indirect immunofluorescence analyses (IFA) using α-Ty antibodies revealed that ASP5-3Ty is a Golgi-resident protein, which co-localizes with the Golgi marker GRASP-YFP. Gliding associated protein 45 (α-GAP45) antibodies were used to stain the parasite periphery. Scale bars represent 2 μm. (B) Western blots completed with α-Ty antibodies, revealed that ASP5-3Ty migrates at the molecular size of 100 kDa, but also as a second lower band of 55 kDa. No Ty signal was detected in *RHΔku80Δasp5* parasites. Complementation with ASP5-Ty gDNA (*asp5g-Ty*) restores the wt pattern while complementation with ASP5-Ty cDNA (*asp5c-Ty*) leads only to production of the 100 kDa band. *T*. *gondii* Actin 1 (α-ACT1) was used as a loading control. (C) In type II parasites, ASP5-3Ty migrates similarly to that seen in type I parasites, with two bands detected at 100 and 55 kDa. No Ty signal was detected for *PRUΔku80Δasp5* parasites. Detection of α-ACT1 was used as a loading control. (D) Intracellular growth type I and type II parasites was assessed after 24 hr in complete media. Parasites lacking ASP5 were not impacted in their ability to replicate intracellularly. Data are mean value ± s.d. of three independent experiments. (E) Deletion of ASP5 resulted in a significant impairment of the lytic cycle, as assessed by plaque formation after 7 days, in both type I and II parasites. Complementation with ASP5-Ty gDNA (*asp5g-Ty)* fully restored plaque formation whereas complementation with ASP5-Ty cDNA (*asp5c-Ty)* led to an intermediate plaque phenotype. (F) Mean area of 10 plaques ± s.d.is depicted. (G) Quantification of BIPPO-stimulated egressed vacuoles. Data show mean ± s.d. of three independent experiments. (H) During natural egress, a significant fraction of the parasites remains trapped either inside the parasitophorous vacuole or the host plasma membrane which resemble detached sphere-like structures. No other apparent phenotype was observed.

ASP5 has previously been described as a Golgi-resident protein when expressed as an epitope-tagged second copy [19]. To determine its role and importance in *T*. *gondii*, we first confirmed the localization of ASP5 by inserting a 3Ty-epitope tag at the carboxyl-terminus of the endogenous *ASP5* locus in both type I (*RHΔku80*) and type II (Prugniaud, *PRUΔku80*) strains. Endogenous ASP5-3Ty co-localizes with the Golgi marker GRASP (Fig 1A) and shows two forms by western blot analyses that have not been previously reported. The 100 kDa band is in agreement with the predicted full length protein size (108 kDa), whereas a smaller form migrates with an apparent molecular weight of 55 kDa (short-ASP5). Markedly, short-ASP5 is not detectable when a cDNA copy of the gene is expressed in the parasites (Fig 1B and 1C). To determine if the short-ASP5 form identified by western blot is the result of a processing event, a pulse-chase experiment followed by co-immunoprecipitation (IP) was performed using anti-Ty antibodies on ^35^S-methionine metabolically labelled ASP5-3Ty expressing parasites. Even during the short pulse, short-ASP5 is readily detectable suggesting that it originates either from an alternative transcriptional initiation, splicing or translational start, but not from a processing maturation event (S1A Fig).

To functionally characterize *ASP5*, knockout mutants were generated in type I and II strains. In *RHΔku80*, two loxP sites were inserted on either side of the *ASP5* coding sequence and the gene was excised by transient expression of Cre recombinase, followed by FACS sorting and cloning. The upstream loxP was inserted along with a KillerRed expression cassette while the downstream loxP was directly fused to a GFP without a promoter as described in S2A Fig. The excised parasites (*RHΔku80Δasp5*) were confirmed by genomic PCR and immunofluorescence analyses (Figs 1A and S2B). In parallel, the CRISPR/Cas9 approach was used to generate frame-shift knockout parasites in type I (*RHΔasp5*) and type II strains (*PRUΔku80Δasp5* and *ME49Δasp5*, with insertion of a selection marker, HXGPRT and DHFR, respectively) (S3A–S3D Fig). In the parental lines *RHΔku80* and *PRUΔku80*, *ASP5* was C-terminally epitope-tagged at the endogenous locus prior to disruption of the gene (S3A–S3D Fig). The frame-shift in *ASP5* induced by CRISPR/Cas9 editing was confirmed by genomic PCR and sequencing as indicated in S3B–S3D Fig.

Plaque assays were performed to assess the importance of ASP5 for parasite fitness over multiple lytic cycles (Fig 1E). *RHΔku80Δasp5*, *RHΔasp5*, and *PRUΔku80Δasp5* parasites formed strikingly smaller plaques compared to the wild-type (wt) parasites and their respective parental non-excised lines, indicating a defect in one or more steps of the lytic cycle (Fig 1F). Morphologically, all of the organelles (inner membrane complex, mitochondrion, apicoplast, rhoptries and micronemes) appeared normal in the absence of ASP5 (S1E Fig). Both type I *RHΔku80Δasp5* and *RHΔasp5* parasites were functionally complemented with either a genomic or cDNA version of wt *ASP5*, or the cDNA coding for the mutated ASP5_D/A_ where the aspartic residue in the first catalytic site (DTG) was converted to alanine (ASP5_D/A_). Complemented parasites expressing either *ASP5* cDNA or gDNA were readily obtained in the absence of positive selection, whereas no transgenic parasites expressing ASP5_D/A_ were obtained unless selection was applied. Although the level of ASP5 expression between endogenously tagged ASP5-3Ty (3 tags) and the complemented strain ASP5g-Ty (1 tag) cannot be directly compared, the full complementation of phenotype by plaque assay with a low level of ASP5g-Ty suggests that the protease is produced in excess in wild type parasites. Interestingly, complementation with *ASP5* gDNA resulted in a complete reversion of the *Δasp5* phenotype in plaque assay, whereas *ASP5* cDNA led to only partial reversion, implying that short-ASP5 contributes to *ASP5* functioning (Fig 1F). In contrast, ASP5_D/A_ failed to complement the *RHΔasp5* phenotype (S1B Fig). Table 1 recapitulates all the *ASP5* modified parasites lines generated in this study.

**Table 1:** *T*. *gondii* strains used and developed in this study.

Strain type	Name	Selection marker	Comments
Type I	*RHΔku80* *	HXGPRT^+^ DHFR^+^	*RHΔku80DiCre* strain abbreviated as *RHΔku80* [49]
	*RHΔku80asp5-3Ty*	HXGPRT^+^ DHFR^+^	C-terminal epitope-tagging at the endogenous locus (3Ty) followed by a loxP site
	*RHΔku80loxPasp5-3Ty*	HXGPRT^+^ BLE^+^ DHFR^+^	Insertion of a loxP site followed by a KillerRed driven by a tubulin promoter, at the 5’UTR of the endogenous locus
	*RHΔku80Δasp5*	HXGPRT^+^ DHFR^+^	Excision by transient expression of Cre-GFP recombinase, FACs sorting
	*RHΔku80Δasp5/asp5g*	HXGPRT^+^ DHFR^+^	pT8-ASP5g-Ty (amplified for cosmid library)
	*RHΔku80Δasp5/asp5c*	HXGPRT^+^ DHFR^+^	pT8-ASP5c-Ty (amplified from cDNA)
	*RH* *	HXGPRT^-^	[56]
	*RHΔasp5*	HXGPRT^-^	CRISPR/Cas9-induced DSB causes frame-shift knockout (stop codon at 390/1012 aa),
	*RHΔasp5/asp5g*	HXGPRT^+^	pT8-ASP5g-Ty (amplified for cosmid library)
	*RHΔasp5/asp5c*	HXGPRT^+^	pT8-ASP5c-Ty (amplified from cDNA)
	*RHΔasp5/asp5c-D/A*	HXGPRT^+^	Dead enzyme complemented strain (DTG mutated to ATG), random integration of pT8-ASP5c-D/A-Ty
Type II	*PRUΔku80* *	HXGPRT^-^ CAT^+^	LDH2-GFP randomly integrated [76]
	*PRUΔku80asp5-3Ty*	HXGPRT^-^ CAT^+^ DHFR^+^	C-terminal epitope-tagging at the endogenous locus (3Ty)
	*PRUΔku80Δasp5*	HXGPRT^+^ CAT^+^ DHFR^+^	CRISPR/Cas9-induced DSB, co-transfection of a HXGPRT cassette flanked by 25 nt homology region
	*ME49* *	HXGPRT^+^	[77]
	*ME49Δasp5*	HXGPRT^+^ DHFR^+^	CRISPR/Cas9-induced DSB, co-transfection of a DHFR cassette flanked by 25 nt homology region

*Strains not generated in this study; DSB: double strand break; aa: amino acid

### Parasites lacking ASP5 exhibit reduced fitness and impaired spontaneous egress

The phenotypic consequences of *Δasp5* were investigated for each step of the lytic cycle. Unexpectedly, intracellular growth assays revealed that all strains examined replicated at a similar rate suggesting that ASP5 does not impact on parasite growth (Fig 1D). To exclude that the rich culture media used here is actually masking a phenotype, we performed an intracellular growth assay in glucose depleted medium. While the control BCKDH mutant parasites were severely slowed, both *RH* and *RHΔasp5* were not impacted under glucose starvation conditions (S1C Fig). Spontaneous egress is a none-synchronous event which cannot be assessed quantitatively, whereas the calcium ionophore A23187 is a strong inducer that tends to mask modest impairments in egress. We were only able to observe a significant defect in egress in both *RHΔasp5* and *PruΔku80Δasp5* parasites when using BIPPO (Fig 1G), a recently described potent inhibitor of phosphodiesterases that triggers egress in apicomplexan parasites [20]. Moreover, during the process of spontaneous egress, a significant fraction of *Δasp5* parasites remained enclosed within a membranous structure, either PVM or host plasma membrane, which possibly delayed infection of new host cells (Figs 1H and S1D, S1 Movie).

### Parasites lacking ASP5 alter the fate of intravacuolar and PVM GRAs and fail to form the membranous nanotubular network

**Fig 2:**
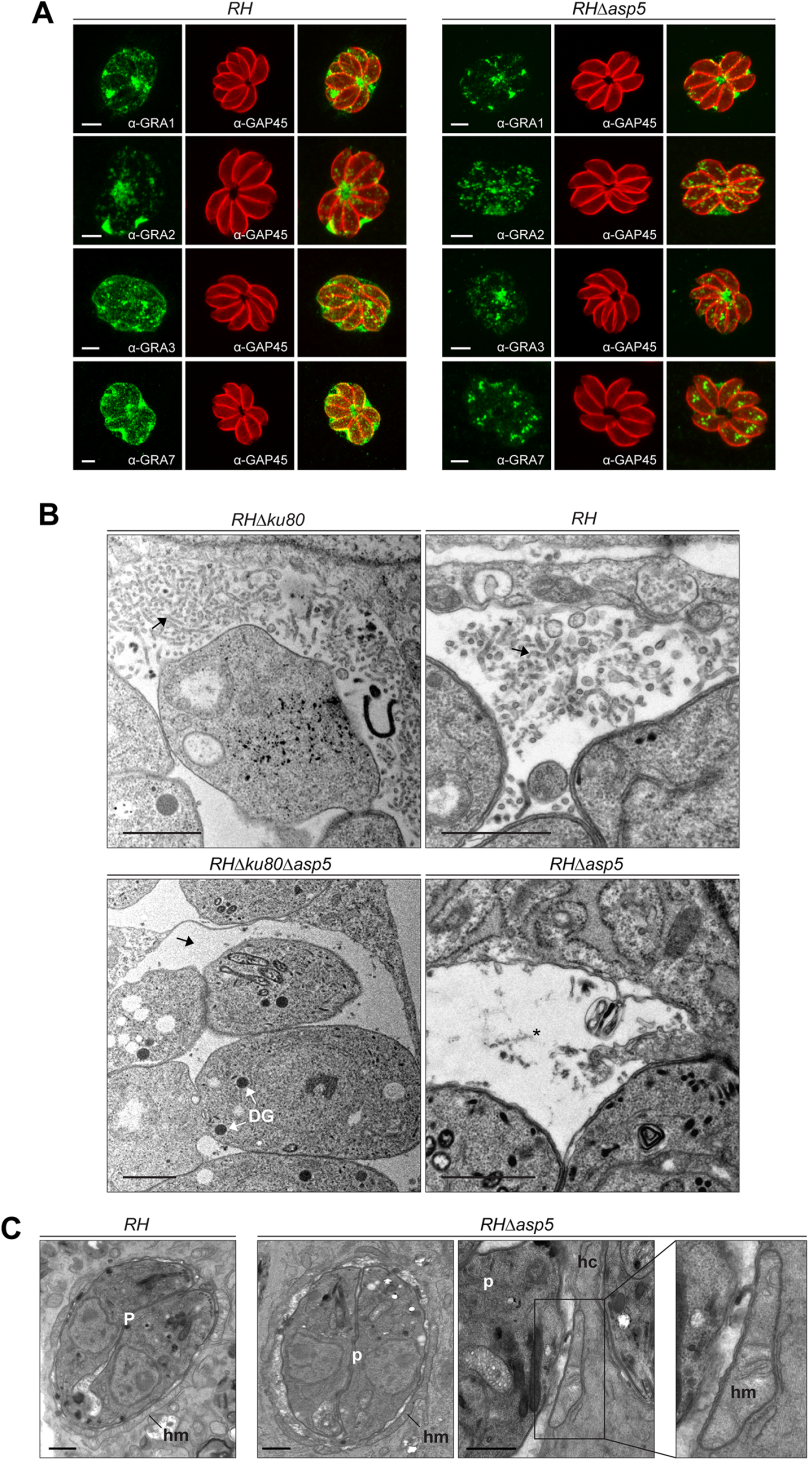
In the absence of ASP5, PV architecture but not host organelle recruitment is altered. (A) In the absence of ASP5 in type I parasites, GRA1 staining was not affected. Conversely, while GRA2 labelling was still present at the PV, it displayed an altered, punctate signal when compared to wt parasites. More strikingly, in the absence of ASP5, GRA3, and GRA7 remained in the vacuolar space and were no longer inserted into the PVM. α-GAP45 antibodies were used to stain the parasite periphery. All IFAs were performed with PFA/GA fixation for 30 min. Scale bars represent 2 μm. (B) Electron micrographs of the membranous nanotubular network (MNN, black arrows) in type I parasites. Strikingly, parasites lacking ASP5 have either no MNN, or occasionally punctate structures (black asterisk) in the vacuolar space. In contrast, ASP5 deletion did not affect the overall ultrastructure of the parasites, particularly the presence of the dense granules (DG). (C) Electron micrographs of infected host cells (hc) showing host mitochondria (hm) association with the PV of *RH* and *RHΔasp5* parasites (p). Scale bars represent 1 μm.

To assess the role of ASP5 in trafficking of the GRAs to the PV and/or the PVM, we first examined the localization of the subset of GRAs for which specific antibodies were available (GRA1, 2, 3 and 7). GRA1 is expressed and secreted into the vacuolar space as a soluble protein that subsequently becomes peripherally associated with the MNN [21]. As shown in Fig 2A, the localization of GRA1, which possesses a putative PEXEL-like motif (RALNK), is not affected by the absence of ASP5. In contrast, GRA2 and GRA3 that are associated with the MNN in wt parasites [13], showed an altered staining pattern in *Δasp5* parasites (Fig 2A). Upon strong fixation conditions adapted to visualize proteins accumulated in the vacuolar space, GRA2 is not aggregated and displays instead a punctate staining for around 80% of the PVs observed. Similarly, GRA7 and also GRA3 localization at the PVM was modified in the absence of ASP5, with no PVM staining observed in more than 70% of the vacuoles (Fig 2A). Given that several GRAs involved in MNN formation appeared perturbed in *RHΔasp5* parasites, the morphology of the PV was examined by electron microscopy. Whilst the MNN in wt parasites is comprised of elongated nanotubules, a dramatic change of vacuolar space architecture was observed in the absence of ASP5. In contrast to *RH* parasites, the PV of *RHΔasp5* parasites did not exhibit a typical MNN which is usually constituted of many long and intricate tubules. Instead, *RHΔasp5* parasites contained vesicles and small tubules sparsely distrubuted throughout the vacuolar space (Figs 2B and S4). This indicates that parasites lacking ASP5 are unable to assemble an elaborated MNN. The PV lumen of *RHΔasp5* parasites also appears different to that of parasites depleted in both GRA2 and GRA6 [13].

By electron microscopy, the PVM appeared intact and the host mitochondria and ER still appeared to be recruited at the periphery of the vacuole (Fig 2C). The outer membrane of the host mitochondria shows a close apposition to the PV membrane of *RHΔasp5* parasites, with a mean distance of 12 ± 3 nm, as similarly documented for *RH* parasites (Fig 2C). Morphometric analyses were undertaken to quantify the extent of host mitochondria-PV membrane association in host cells 24 h p.i.: 26 and 18% of the PV membrane was associated with host mitochondria in *RH* and *RHΔasp5* parasite-infected cells, respectively. This suggests that the mutant has the ability to recruit host mitochondria to its PV but to a lesser extent than wt parasites.

### ASP5 is necessary for cleavage of GRA19 and GRA20 but does not affect GRA secretion to the PV

**Fig 3:**
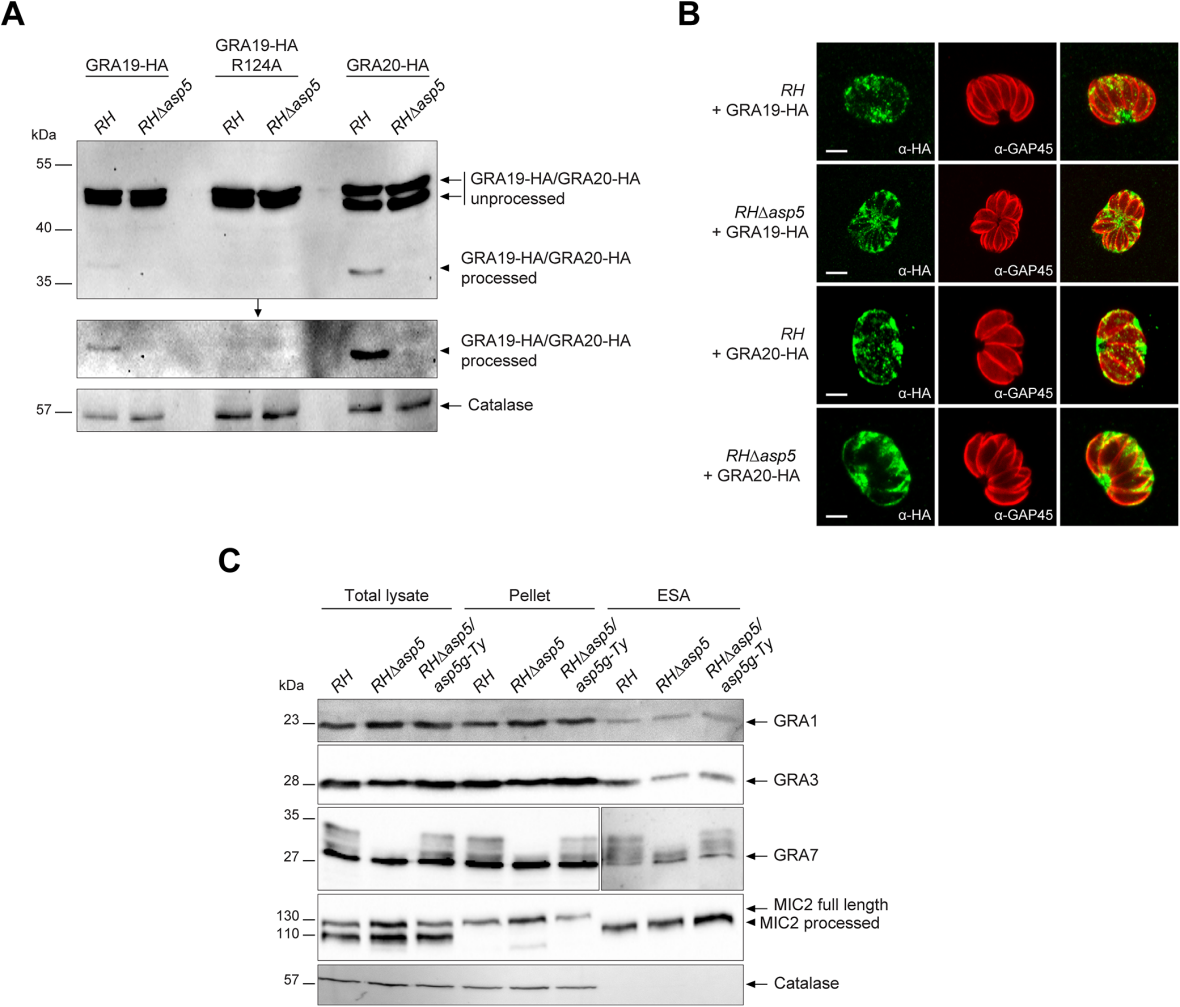
ASP5 mediates the cleavage of PEXEL-like motif-containing proteins but does not impact on dense granule secretion. (A) Type I parasites were transiently transfected with GRA19-HA, GRA19-HA-R124A (PEXEL-like mutant control) or GRA20-HA. Intracellular parasites were collected and analysed by western blot. In the absence of ASP5, processing of GRA19-HA and GRA20-HA was abolished as detected by α-HA antibodies. Middle gel represents a longer exposure. α-Catalase antibodies were used as a loading control. (B) IFAs of parasites transiently transfected with GRA19-HA and GRA20-HA as presented in (A). The absence of the ASP5-dependent processing of GRA20 did not affect its localization at the PVM. Scale bars represent 2 μm. (C) Dense granule secretion assays in type I parasites revealed that deletion of ASP5 does not impair GRA1, 3 or 7 secretion. Interestingly, the multiple band pattern of GRA7 is abolished under these conditions. Catalase and MIC2 (micronemal protein 2) were used as controls for the non-secreted and secreted fraction respectively (MIC2 is processed upon secretion of the micronemes). ESA: excretory secretory antigens.

The recently described GRA19 and GRA20 were investigated here via expression of C-terminally HA-tagged second copies as previously described [18]. Both proteins are known to be processed within their PEXEL-like motif by an unidentified protease [18]. Transiently expressed GRA19-HA and GRA20-HA were modestly processed as previously observed in parental parasites, however this cleavage was abolished in the absence of ASP5 (Fig 3A). An R/A point mutant in the GRA19 PEXEL motif prevented processing as previously reported, and served here as a control. The absence of ASP5 did not alter the localization of either GRA19 or GRA20 in an obvious manner as documented by IFA (Fig 3B). Given that both processing and localization of several GRAs is affected by the absence of ASP5, we examined whether the overall secretion by dense granules was impaired. We developed a secretion assay whereby released GRAs were collected from the supernatant of extracellular parasites and referred to here as excretory secretory antigens (ESA) upon western blot analyses. Secretion of the microneme protein MIC2 was used here as a control for parasite viability and fitness. These assays revealed that secretion of GRA1, 2, 3 and 7 were comparable in *RH* and *RHΔasp5* strain parasites (Fig 3C). Interestingly, GRA7 which was previously reported to be phosphorylated by an unidentified host cell kinase [22, 23] gave rise to a ladder of bands which appears to be extensively reduced or even abolished in the absence of ASP5 (Fig 3C). This suggests that GRA7 may be subtly miss-targeted in the absence of ASP5 and hence no longer accessible to the host kinase. Taken together, these results indicate that ASP5 is responsible for the cleavage of some PVM-enclosed GRAs and in its absence, these proteins are normally secreted by the dense granules yet are impacted in their final destination. This is likely to lead to defects in post-translational modifications (e.g. phosphorylation of GRA7) and altered protein activity given that the MNN is no longer formed.

### ASP5 is necessary for the export of the extravacuolar GRAs

**Fig 4:**
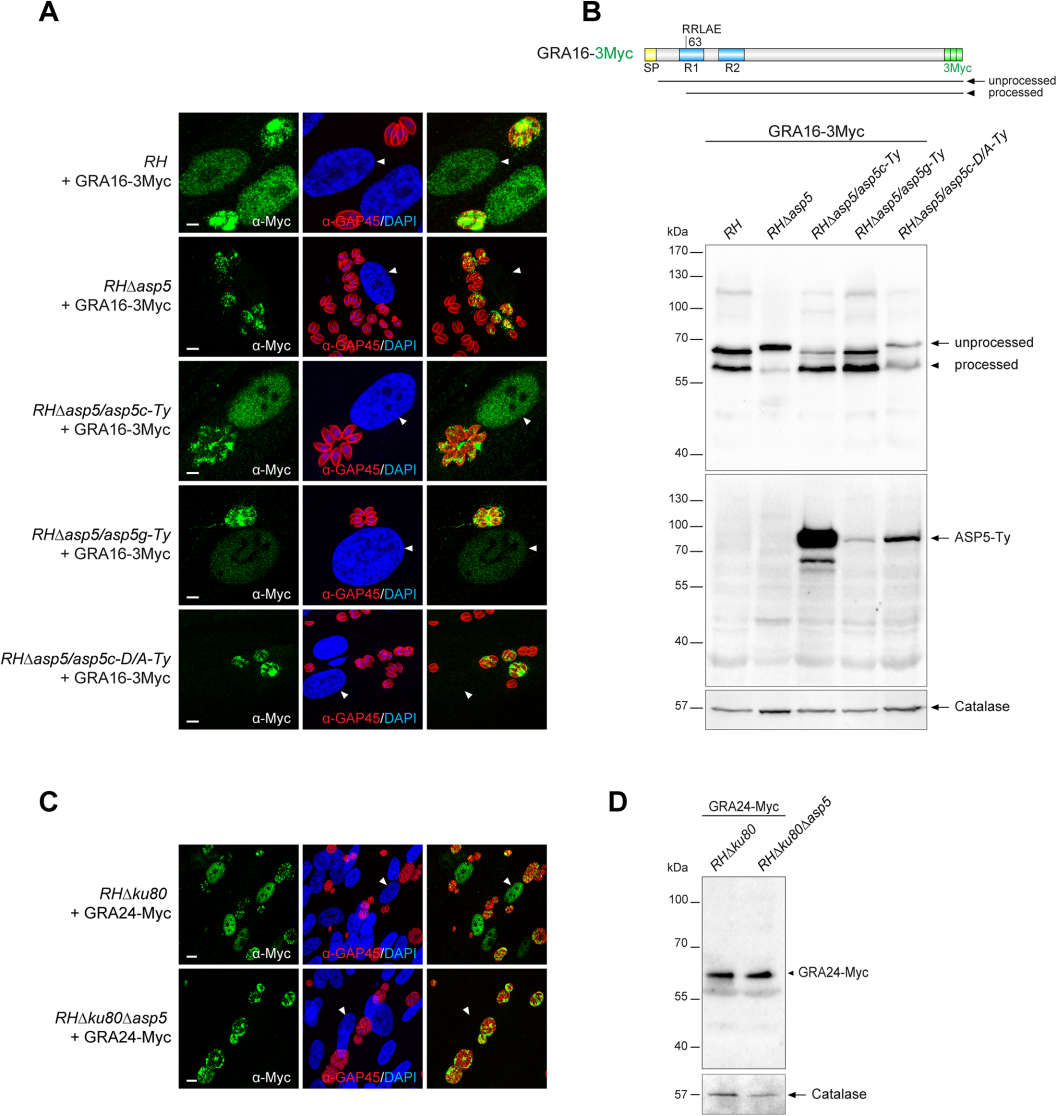
ASP5 deletion compromises the export of GRA16 and GRA24 beyond the PVM. (A) In the absence of ASP5, GRA16-3Myc transiently transfected in type I parasites was no longer addressed to the host nucleus (white arrowheads) and instead remained within the PV. Scale bars represent 2 μm. (B) The PEXEL-like motif-containing GRA16-3Myc displays an altered cleavage profile in *RHΔasp5* and *RHΔasp5*/*asp5c-D/A* parasites as detected by western blot analyses. R1 and R2: repeated region. (C) IFAs of a stably expressed second copy of GRA24-Myc revealed that, in parasites lacking ASP5, this protein was no longer addressed to the host nucleus and instead accumulates within the PV. (D) GRA24-Myc, which is devoid of PEXEL-like sequence, shows a similar migration profile in both strains. For panel B and D, parasites were collected 24 hr post-infection. α-Catalase was used as a loading control.

Following parasite internalization and the concomitant PVM formation, two GRAs are known to cross the PVM and be exported into the host cell nucleus [16, 17]. To assess the fate of GRA16 and GRA24, second copies of GRA24 driven by a tubulin promoter and GRA16 driven by its endogenous promoter and fused to 3 Myc tags were expressed in type I parasites (Fig 4A and 4C). As previously reported, GRA16 and GRA24 show a dual localization in the PV as well as the host cell nucleus in *RH* parasites. In sharp contrast, in *RHΔasp5* parasites both GRAs accumulate in the PV but fail to reach the host nucleus, even at a high MOI. Whereas *ASP5* cDNA or gDNA complementation restored the host cell nuclear localization, the catalytically inactive ASP5_D/A_ is not sufficient to promote GRA16 export (Fig 4A). In wild type parasites GRA16-3Myc showed two forms that presumably correspond to unprocessed and processed forms given the fact that the protein possesses a PEXEL-like motif starting at the arginine residue in position 63, corresponding after cleavage to a drop of ~5 kDa. Contrastingly, in *RHΔasp5* parasites the unprocessed form of GRA16 (which migrated slightly slower) strongly accumulates while a residual level of the processed form was still detectable (Fig 4B). This might result from the action of a different protease, or may represent a degradation product. The small shift in the unprocessed band observed between *RH* and *RHΔasp5* parasites cannot be explained and will require further investigation.

Scrutiny of the GRA24 sequence did not uncover the presence of such a motif, nor did the protein appear to undergo any detectable processing event by western blot analyses (Fig 4D).

These findings demonstrate that ASP5 contributes critically to the export of parasite effector proteins both with and without a PEXEL-like motif suggesting that ASP5 might additionally alter the function of protein(s) implicated in the translocation of effectors across the PVM.

### Type I and type II parasites lacking ASP5 are less virulent in the mouse model

**Fig 5:**
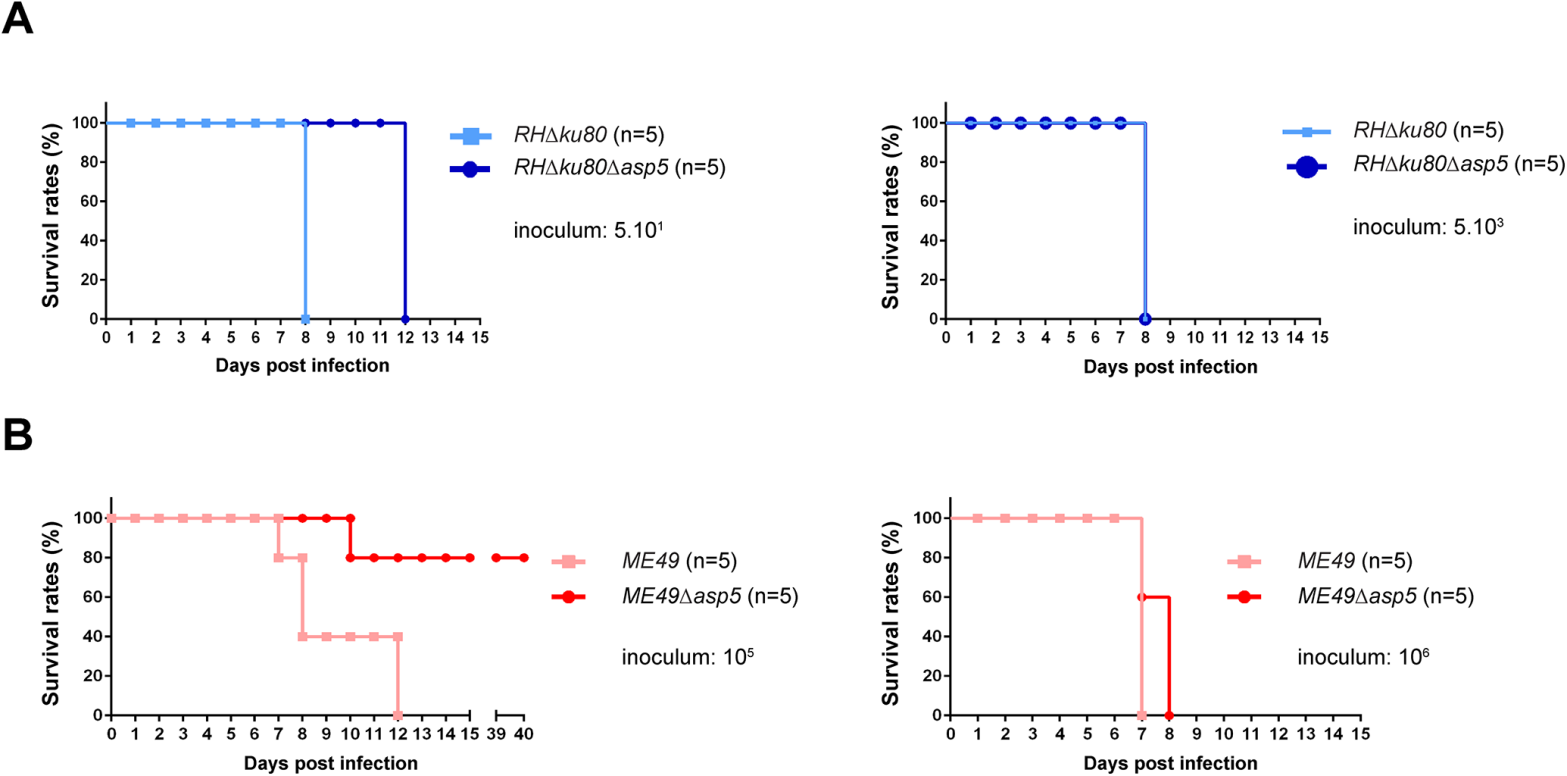
ASP5 deletion affects *in vivo* virulence of type I and type II parasites. (A-B) C57Bl/6 mice were inoculated by intraperitoneal injection of 5.10^1^ or 5.10^3^ (A) type I parasite, and 10^5^ or 10^6^ (B) for type II parasites. Mice survival was monitored over time. Five animals were infected for each experiment.

Type I parasites lacking GRA16 and GRA24 exhibit no decrease in virulence in mice, however the deletion of these genes in type II strain parasites has been reported to show reduced virulence [16, 17]. In light of this, type I and type II *Δasp5* parasites and their parental lines were assessed for virulence upon intraperitoneal (i.p.) inoculation into groups of susceptible female C57Bl/6 mice. Mice had to be sacrificed 7 days after infection with 5.10^1^ *RH* parasites, whereas mice receiving the same inoculum of *RHΔasp5* parasites survived for 13 days (Fig 5A). Despite this, upon inoculation of a larger number of parasites (5.10^3^) no difference was observed between the two type I parasite lines (Fig 5A). This suggests that the delay observed during low dose infection could be explained by the reduced fitness observed in tissue culture. Mice infected with 10^6^ type II parasites from both *ME49* and *ME49Δasp5* led to death of the animals over the acute phase of the infection (Fig 5B). In contrast, 80% of the mice infected with 10^5^ *ME49Δasp5* survived the infection at day 40 whereas the control *ME49* parasite line succumbed to infection within 7 to 13 days (Fig 5B). The type II *Δasp5 in vivo* phenotype therefore correlates with previous observations made with GRA16 and GRA24 deficient parasites [16, 17]. Seroconversion was assessed for the four surviving mice, which all display a positive serological profile (S5 Fig). Cyst biogenesis *in vivo* remains unassessed and will be further investigated.

### Type I parasites lacking ASP5 are still able to neutralize the IRG-based host cellular defense mechanism

**Fig 6:**
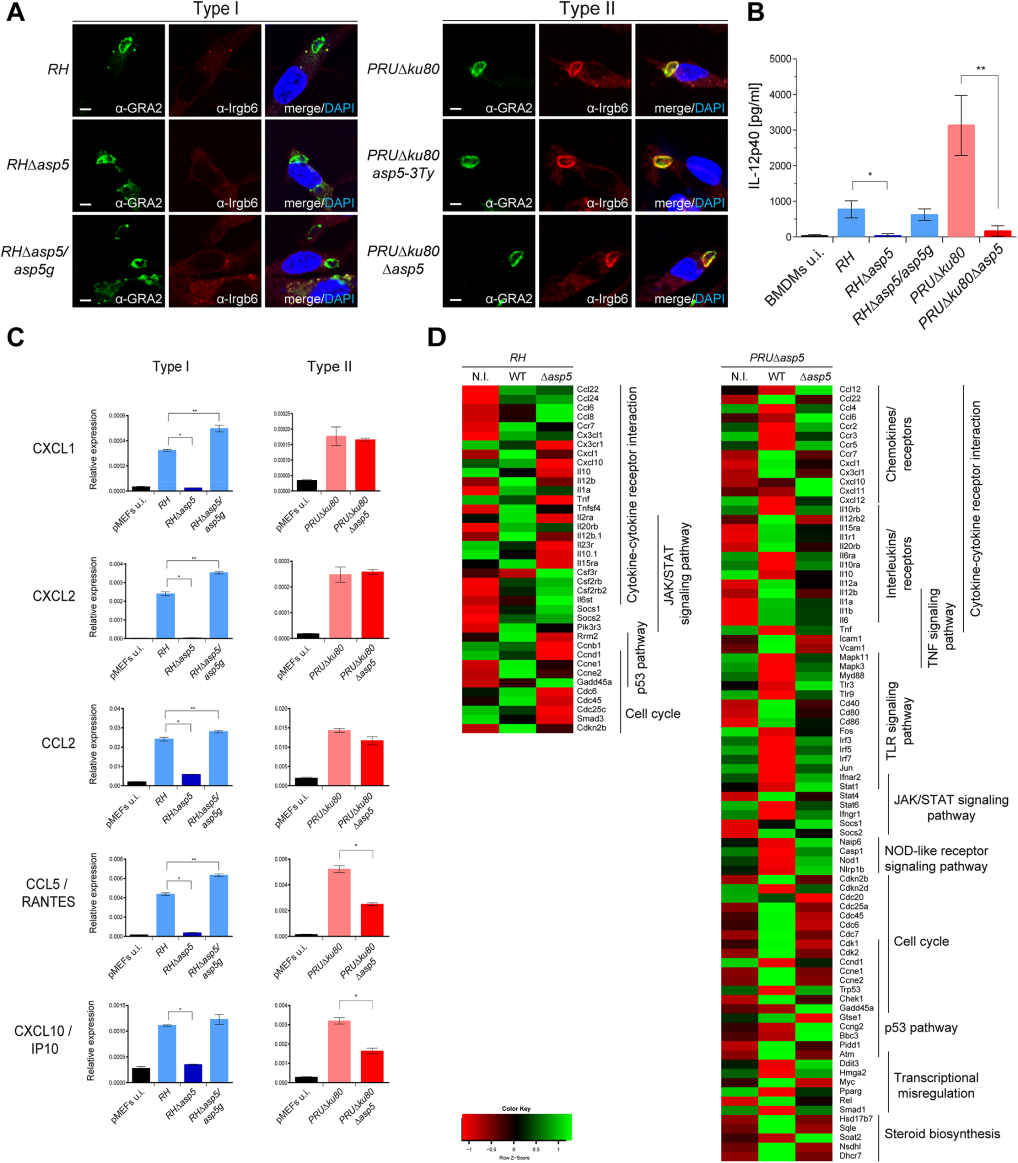
ASP5 modulates host innate immune responses. (A) Interferon-γ stimulated, bone marrow derived macrophages (BMDMs) were infected with type I and type II parasite lines for 1 hr at a MOI of 1. In the absence of ASP5, type I parasites were not affected in IRG recruitment to the PVM. Type II parasites were used as controls. Parasites were labelled with α-GRA2, host IRGs with α-Irgb6 antibodies, and host nuclei with DAPI. IFAs are representative data from three independent biological experiments. Scale bars represent 2 μm. (B) IL-12p40 levels were measured by ELISA from supernatant collected 40 hr after BMDM infection with type I and type II strain parasites. Supernatant from uninfected cells was used as a control (u.i.). In both types, the absence of ASP5 dramatically reduces IL-12p40 levels. Data are mean value ± s.d. of three independent experiments. (*P<0.01, **P<0.005, Student’s *t*-test). (C) Quantitative chemokine production was determined by qRT-PCR on mouse embryonic fibroblast (pMEFs) infected with type I and type II parasites. Values were normalized to the amount of actin in each sample. Data are mean value ± s.d. of three independent experiments. (*P<0.01, **P<0.05, Student’s *t*-test). (D) Following KEGG analysis of the differentially expressed genes (>2 fold, p-value < 0.05) between parental and *Δasp5* infected BMDMs, heatmaps were generated with genes selected from representative KEGG pathways. (Red: low, Green: high). Both up- and down-regulated genes of the host cell were analyzed together.

One of the key events in establishing a protective Th1 immune response against *T*. *gondii* is the ability of host immune cells to produce the pro-inflammatory cytokine interleukin 12 (IL-12), which in turn stimulates the production of interferon gamma (IFNγ) by natural killer (NK) cells, CD4+ and CD8+ T cells [15, 24]. IFNγ is the major pro-inflammatory cytokine driving multiple cellular defense mechanisms during both the acute and chronic phases of infection [25]. Importantly, the immunity related GTPases (IRG proteins) constitute a large family of interferon-inducible proteins that mediate early resistance to *T*. *gondii* infection in mice. Several studies have shown that IRGs, in particular Irga6 and Irgb6 are recruited to the nascent PVM, where they cause disruption of the vacuole and parasite death. While the ROP18 complex of type I parasites is able to phosphorylate IRG proteins, thereby preventing their oligomerization and loading onto the PVM, type II parasites are unable to block the action of IRG proteins due to the polymorphic nature of ROP5, which forms part of the ROP18 complex [26]. Recent studies have associated GRA7 to the ROP18 complex by acting as regulator for ROP18-specific inactivation of Irga6 [23, 27]. To determine whether vacuoles containing *RHΔasp5* parasites failed to block IRG recruitment to the PVM, an IRG recruitment assay was performed for Irgb6. Our results indicate that *RHΔasp5* parasites behave like *RH* parasites and remain non-susceptible to Irgb6 and Irga6 loading (Fig 6A, left panel). Conversely, Irgb6 was recruited to the PVM of *PRUΔku80Δasp5* parasites as previously reported for *PRUΔku80* (Fig 6A, right panel) [26]. Given these data and in spite of the impact of ASP5 on GRA7 phosphorylation (which forms part of the ROP18 complex), we propose that ASP5 activity is not essential for the activity of the ROP18 complex [23].

### Type I and type II parasites lacking ASP5 are defective in modulating the host immune response to infection

In light of the blockage in the export of GRA effectors, we interrogated the impact of *Δasp5* on the macrophage response to infection. Differentiated bone marrow derived macrophages (BMDMs) were infected with type I and II strain parasites lacking ASP5, and IL-12p40 levels were measured 40 h pi (at the peak level of IL-12p40 secretion) by ELISA. As previously observed, *PRU* parasites induce significantly higher levels of IL-12p40 synthesis when compared to *RH* strain parasites (Fig 6B). Both type I and type II strain parasites showed a reproducible and significantly lower level of IL-12p40 produced by macrophages infected with *Δasp5* parasites. Expectedly, *RHΔasp5/asp5g* complemented parasites rescued the IL-12p40 phenotype observed with *RHΔasp5* parasites. This assay has also been carried out with RAW264.7 cells and with peritoneal exudate cells (PECs), and both assays produced results comparable to those obtained with BMDMs. Based on the current knowledge, both type II GRA15 and GRA24 promote IL-12 secretion *in vitro* and *in vivo* and it is therefore plausible that export and function of GRA15 is also blocked in the absence of ASP5 [15].

Chemokines are soluble mediators that are essential to contain parasite spreading and to control the infection. Previous studies have shown that *T*. *gondii* induces chemokine up-regulation in several cell types and specifically GRA6 [28], GRA24 [17] and GRA25 [29] are known to shape the immune response by regulating the expression of CXCL1, CXCL2, CCL2, CCL5 and CXCL10 [30]. Here, monolayers of pMEF cells were infected with type I and type II strain parasites and pelleted after 20 h. cDNA was synthesized from total RNA and the mRNA expression levels for each of these chemokines was measured by qPCR. These data reproducibly demonstrated a pronounced decrease in CXCL1, CXCL2, CCL2, CCL5 and CXCL10 expression reproducibly measured from cells infected with *RHΔasp5* parasites when compared with *RH* and *RHΔasp5/asp5g* parasites (Fig 6C). The results obtained with *PRU* and *PRUΔasp5* showed no difference for CXCL1, CXCL2 and CCL2, while a significant decrease in CCL5 and CXCL10 expression was observed.

To obtain a more global picture of the modification caused by the absence of ASP5, we performed a genome-wide expression profiling by RNA-sequencing of mouse BMDMs infected with parental or *Δasp5* parasites from both type I and type II parasites (Fig 6D). We focused our analysis on genes that were modulated with more than twofold change when comparing each *Δasp5* mutant with their respective parental strains. A broader, analysis of the highly modulated KEGG pathways in a type I context of infection revealed that a substantial number of both pro- (e.g., *IL-1a* and *TNF*) and anti-inflammatory (e.g., *IL-10*) cytokines, chemokines and their relative receptors (e.g., *CXCL1*, *CXCL10*) were significantly differentially-regulated upon *Δasp5* parasite infection compared to parental lines (Fig 6D, left panel). Strikingly, in type II *Δasp5* parasites, the parasite-induced transcriptional response is strongly reduced to a level resembling un-infected cells (Figs 6D, right panel, S6A and S6B). This result highlights that ASP5 might broadly impact type II-specific virulence factors and host cell effectors.

### Parasites lacking ASP5 are hampered in their ability to trigger dendritic cell hypermigration

**Fig 7:**
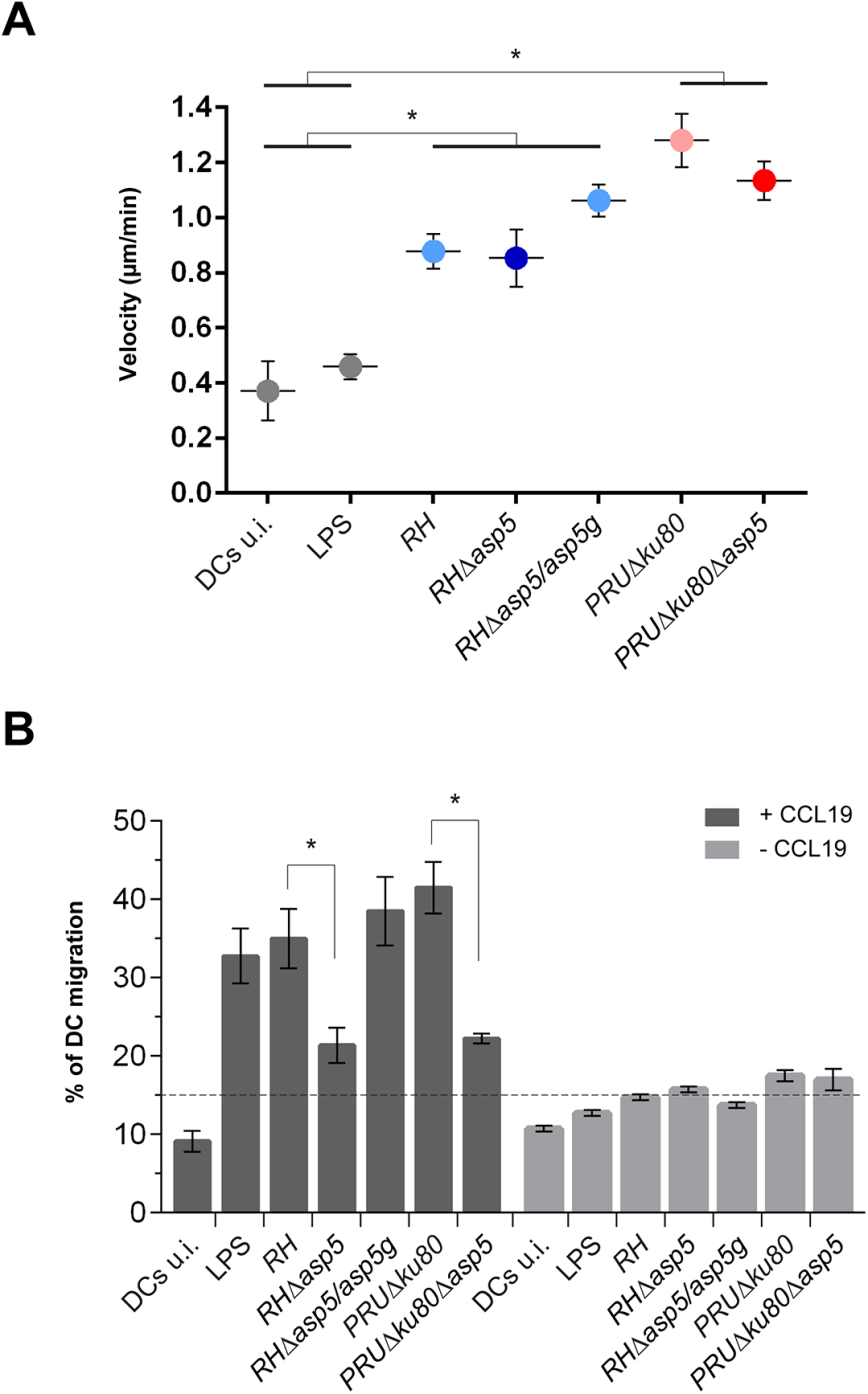
ASP5 affects dendritic cell (DCs) transmigration but not hypermotility. (A) Cell track analyses of DCs were assessed 6–8 hr post-infection for each condition. Circles represent average speed (μm/min) ± s.d. of 100 single cells measured during 45 min from one representative experiment (*P<0.01, Student’s *t*-test). (B) Transmigration of DCs was assessed in a transwell system +/- CCL19 16 hr post-infection with either type I or type II parasites. CCL19 induction resulted in the transmigration of wt or complemented parasites whereas in *Δasp5* strains, transmigration was reduced almost to the same level as the non-induced samples. The graph shows one representative condition ± s.d. from three independent biological replicates (*P<0.01, Student’s *t*-test).

*T*. *gondii* tachyzoites can cross biological barriers [31, 32], however, once inside the host the precise mechanisms leading to systemic dissemination of the parasites remain unknown. *T*. *gondii* can exploit the migratory properties of dendritic cells (DC) to spread throughout the organism using the “Trojan horse” strategy. Specifically, upon infection by tachyzoites, DCs exhibit a hypermigratory phenotype [33, 34]. GRA5 has been described as one of the parasite effector molecules capable of increasing the migratory properties of DCs via CCR7 expression without DC activation [35]. To determine the potential impact of ASP5 in this process we first assessed the hypermotility phenotype and observed no significant differences between *Δasp5* and the corresponding parental lines either in *RH* or *PRU* parasites (Fig 7A). We then conducted a transmigration assay, whereby we measured the ability of infected DCs to migrate in response to CCL19, a CCR7 ligand [34]. In this assay both *RHΔasp5* and *PRUΔku80Δasp5* parasites showed a considerable reduction in transmigration of infected DCs (Fig 7B).

### Cyst wall formation

**Fig 8:**
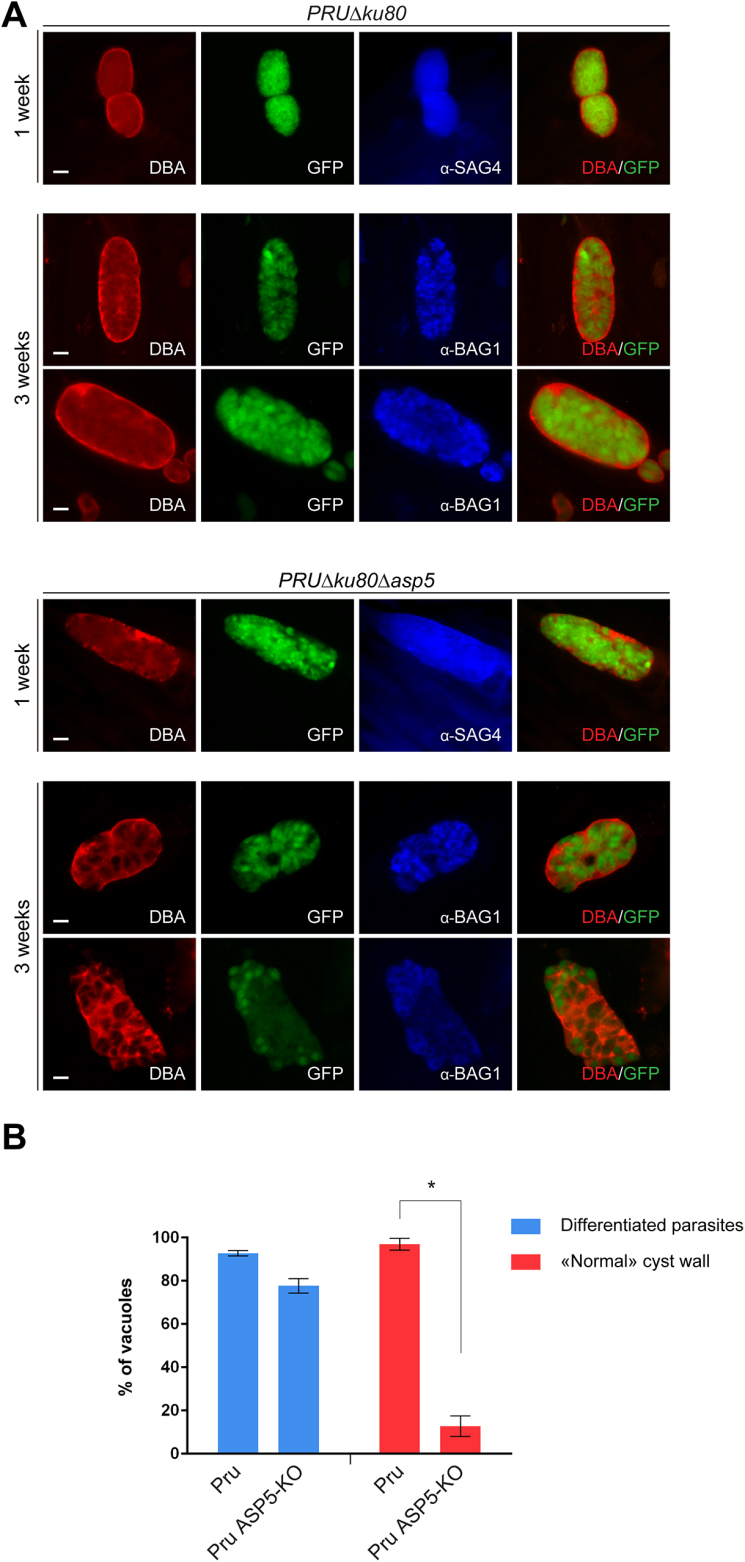
Cyst wall formation is affected in type II parasites lacking ASP5. (A) Induced bradyzoite differentiation of type II parasites under high pH conditions. In parasites lacking ASP5, cyst wall formation, as visualized with DBA lectin, was already affected 1 week after induced-differentiation. α-SAG4 (surface antigen 4) and α-BAG1 (bradyzoite antigen 1) were used as bradyzoite markers. GFP is under the control of a bradyzoite lactate dehydrogenase 2 (LDH2) promoter. Scale bars represent 2 μm. (B) Quantification of the representative IFAs depicted in panel (A). Data are mean value ± s.d. of three independent experiments.

Importantly, GRAs are anticipated to play key roles in other stages of the parasite and notably during cyst wall formation, a process that is central for parasite persistence and transmission [36]. To assess the role of ASP5 in cyst wall formation, we used the fluorescent *Dolichos biflorus* Agglutinin (DBA) lectin to detect the glycosylated protein CST1 (one of the few available markers of the *T*. *gondii* cyst wall) [37], following pH induced differentiation of *PRUΔku80Δasp5* strain parasites *in vitro*. Stage conversion from tachyzoites to bradyzoites was measured by expression of SAG4 or BAG1. This conversion took place normally in *PRUΔku80Δasp5* parasites and CST1 was also produced, glycosylated and targeted to the PV However, upon closer inspection of the IFA, it became obvious that cyst wall formation was already impaired just one week after induction of differentiation, and even more strikingly impaired two weeks later (Fig 8A and 8B).

## Discussion

Whilst intracellular, apicomplexan parasites reside within a specialised membranous niche (PVM), across which the parasite transports a plethora of effector molecules necessary for subversion and remodelling of host cell functions. Studies in *P*. *falciparum* have revealed that this process relies upon a PVM-resident translocation machinery (PTEX) that serves to facilitate export of parasite proteins across this membrane into the erythrocyte cytosol [38, 39]. Intimately associated with this process is Plasmepsin V, a protease known to cleave a specialised motif (PEXEL) in a wide repertoire of known exported proteins [40–42]. Similarly, *T*. *gondii* possesses components related to the PTEX translocon that are proposed to act as a molecular sieve at the PVM, allowing diffusion of small molecules across this membrane [43].

**Fig 9:**
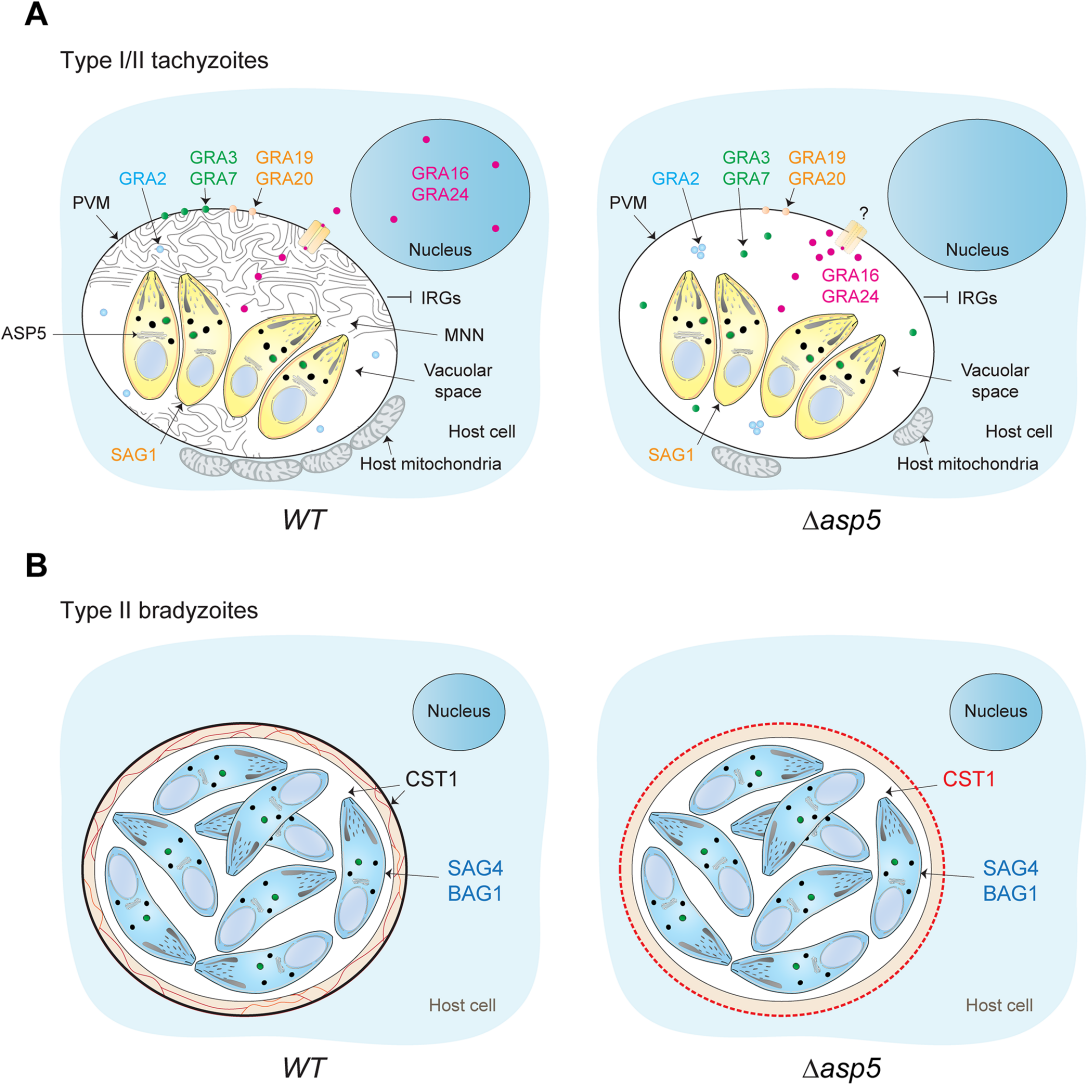
Stage specific functions of ASP5 in *T*. *gondii*. (A) Schematic representation of a host cell infected by either type I or type II tachyzoites. In a wt situation (left panel), intracellular tachyzoites reside within an MNN containing PV. During intracellular development, dense granule proteins are delivered either to the vacuolar space, the PVM, or are addressed to the host cell cytosol and nucleus. Intriguingly, the absence of ASP5 (right panel) does not affect the secretion of the dense granules, and accordingly, PEXEL-like and non-PEXEL-like motif-containing proteins are still delivered to the PV. However, ASP5 deletion leads to the disappearance of the MNN, altered localization of some PV- and PVM-resident GRAs, and to the PV-accumulation of proteins normally exported to the host cell cytosol. Accordingly, the overall infected host cell transcription profile response is significantly different in *Δasp5* parasites infection. The accumulation of some exported GRAs in the PV suggests that the export machinery used to cross the PVM is affected upon ASP5 deletion. In contrast, no significant effect was observed for early host organelle recruitment to the PVM, but only a reduced fusion of host mitochondria was observed. Specifically in type I parasites, blocking of IRG recruitment is unaffected. SAG1 is a tachyzoite marker. (B) Differentiation in bradyzoites is not affected by ASP5 deletion but parasites fail to form a cyst wall. SAG4 and BAG1 are bradyzoite markers.

Here we report the characterisation of the Golgi-resident protease ASP5, which is responsible for the cleavage of PEXEL-like motif-containing proteins in *T*. *gondii*. Whilst Plasmepsin V appears to be primarily dedicated to cleavage of proteins destined to be exported beyond the PVM, deletion of ASP5 causes considerable pleiotropic effects by effecting both exported and PV/PVM-resident proteins (Fig 9). Accordingly and in contrast to *P*. *falciparum*, numerous PEXEL-like containing proteins remain within the PV and are not further exported. Deletion of ASP5, without affecting dense granule secretion, caused significant morphological aberrations of the PV, most notably being the defect in MNN formation. The role of this elaborated structure is still mysterious, although it is presumed to participate in parasite access to host cell nutrients. In this context, and rather unexpectedly, depletion of ASP5 does not appear to impose any restriction on intracellular parasite replication even in glucose depleted media. The molecular connection between ASP5 activity and MNN formation is not known, however such a phenotype was previously described when individual GRAs were knocked out [13]. Alternatively, the MNN could participate in the process of egress, which is unexpectedly affected in parasites lacking ASP5. It is known that some GRAs form high molecular weight complexes within the dense granules [44] and also exist as heteromeric complexes in the PV [44]. It is therefore conceivable that deletion of ASP5 could affect formation of these complexes and thus ASP5 will not only affect the activity of its direct substrates but also their interacting partners.

Through western blotting analyses we demonstrate that the vacuolar GRA19 and GRA20, together with the exported GRA16, are cleaved by ASP5 at the PEXEL-like motif. These proteins share a consensus motif “RRL” in their PEXEL-like sequence, which is likely part of a larger motif recognized and cleaved by ASP5. This slightly differs from the substrate preference of Plasmepsin V [45], and may suggest that the motif serves differing functions in *T*. *gondii*.

In wt parasites, multiple GRAs such as GRA16 and GRA24 are exported to the host cell nucleus. Upon ASP5 deletion however, there is a striking block in the export of GRA16 and GRA24 and they are absent in the host nucleus but still accumulated within the PV. Intriguingly, while GRA16 harbours a PEXEL-like motif and thus accumulates as an un-cleaved product in the absence of ASP5, GRA24 is apparently devoid of such a signal. Assuming that both GRA16 and GRA24 share the same translocation pathway to cross the PVM, the defect in GRA24 export suggests that a component of the export machinery is defective and hence possibly active only upon cleavage by ASP5. A recent study has demonstrated that ASP5 is directly responsible for the processing of GRA16 in a PEXEL-dependent manner at the site (RRLAE) [46]. In accordance with our findings, this study established that the mapped processing of GRA16 is actually not necessary for export of the protein to the host nucleus. Further dissection of GRA16 processing and identification of components of the translocation machinery and the potential implication of ASP5 in this process still await further investigation.

The aforementioned GRAs exported to the host cell nucleus play a significant role in reprogramming host cell gene expression and thus contribute to immunomodulation of the host. This response is significantly perturbed in parasites depleted of ASP5 as reflected by the targeted analysis of IL-12 and the chemokine levels, as well as by the global transcriptome analysis of BMDMs infected with either type I or type II parasites.

Previous studies in type II strain parasites reported specific host-transcriptome alterations when selected GRAs [15–17, 29] were knocked-out individually. While reproducing most of these findings our data significantly broaden the network of putative parasite-dependent host-regulated pathways. Importantly, the broader impact observed upon ASP5 deletion reflects the multiple GRAs affected simultaneously in this mutant. *T*. *gondii* also acts to subvert host cell functions through increasing the motility and migration of infected DCs. This parasite-induced hypermigration of infected DCs ensures rapid dissemination of the parasites from the intestinal site of entry to the rest of the body. This step is critical for the establishment of infection and persistence as it gives the parasite time to reach sanctuary organs prior to the onset of the immune response [47]. The mediators of DCs hypermigration are not fully characterized; however GRA5 has been reported to be associated with this phenomenon [35]. Parasites lacking ASP5 show a considerable loss of this capacity, which correlates with the down-regulation of CCR7 observed in the RNA seq data upon *Δasp5* type I and type II parasite infection.

The establishment of chronic infection is underpinned by cyst formation. Not only do cysts ensure transmission from intermediate to definitive hosts, they are also the source of reactivation in situations of immunosuppression and thus a very important stage from a pathology view-point. Our data indicate that whilst ASP5 does not impact upon the capacity of the parasite to differentiate into bradyzoites, depletion of ASP5 severely compromises the parasites ability to build the cyst wall. The cyst wall provides a protective ‘shell’ within which the parasite is sheltered from the host cell environment, however exchanges across this barrier with the host cell are still anticipated to occur.

In conclusion, the role of the *T*. *gondii* PEXEL-like motif appears to be significantly broader than that reported to date for *Plasmodium spp*., wherein PEXEL cleavage gives rise to a newly exposed N-terminal sequence proposed to serve as a trafficking signal for proteins destined to be exported beyond the PVM. Given the substantial differences in host cell repertoire and accordingly, the host cell subversion requirements of these two apicomplexan parasites, it is not surprising that cleavage of this motif in *T*. *gondii* is likely implicated in a variety of functions. For example, the ASP5 PEXEL-like motif cleavage could be needed to elicit conformational changes needed for enzymatic activity, or alternatively, for sequential interaction/recognition by/with a host/parasite partner. In the context of protein targeting, given that *T*. *gondii* PEXEL-like motif cleavage occurs within the Golgi prior to trafficking to the DGs, it might specifically target a population of proteins to distinct secretory organelles. Concordantly, not all exported GRAs completely co-localize with canonical GRA-markers within the parasite [16, 17]. Whilst the data presented here have substantially contributed to the knowledge base surrounding not only ASP5 but also GRA functioning, many questions pertaining to the reasons behind this cleavage remain to be answered. Furthermore, we have not estimated the range of ASP5 substrates. Notably, the option that ASP5 mediated cleavage could expand to proteins from secretory organelles other than dense granules or following the default pathways for secretion, has not been comprehensively investigated. In spite of this, this study will serve as a solid platform upon which further investigations into the essential process of apicomplexan protein export can be completed.

## Materials and Methods

### Bacteria, parasite strains and host cell culture

*E*.*coli* XL-10 Gold chemo-competent bacteria were used for all recombinant DNA experiments. *T*. *gondii* tachyzoites parental and derivative strains were grown in confluent human foreskin fibroblasts (HFFs) maintained in Dulbecco’s Modified Eagle’s Medium (DMEM, Gibco) supplemented with 5% fetal calf serum (FCS), 2mM glutamine and 25 mg/ml gentamicin.

### Cloning of DNA constructs

Genomic DNA (gDNA) from RH parasite was isolated with the Wizard SV genomic DNA purification system (Promega). Total cDNA was generated by RT-PCR using the Superscript II reverse transcriptase (Invitrogen). TgASP5 ToxoDB accession number: TGME49_242720. The C-terminal (Ct) of ASP5 was amplified with primers 4203–4204 on gDNA and cloned in pT8-TgMIC13-3Ty-HXGPRT [48] between *ApaI* and *NsiI* sites to give Ct-ASP5-3Ty-HXGPRT. This vector was then digested *ApaI*/*PacI* and cloned in pTub8-loxP-KillerRed-loxP-YFP-HXGPRT [49] to give Ct-ASP-LoxP-YFP-HXGPRT. The 5’ region of ASP5 was amplified with primers 4615–4616 and cloned in pTub8-loxP-KillerRed-loxP-YFP. This vector was digested *PacI*/*SacII* and the bleomycin selection cassette from pTub8-ARO-GFP-Ty-Ble [50], digested with the same sites and was inserted to give 5’ASP5-pTub8-loxP-KillerRed-Ble. Ct-ASP5-3Ty-HXGPRT was digested *ApaI*/*NotI* and ligated into p2854-DHFR-TS [51] to create Ct-ASP5-3Ty-DHFR. To create pTub8-ASP5c-Ty, cDNA was amplified with primers 1624–1592, digested *MfeI*/*NsiI* and cloned in pTub8-Ty [52] digested *EcoRI*/*NsiI*. To create pTub8-ASP5g-Ty, cosmid PSBL804 (D. Sibley, Toxodb.org) was digested *BglII*/*EcoRI*, the band corresponding to the genomic DNA of ASP5 was isolated and cloned at the same sites in pTub8-ASP5c-Ty. Importantly, the last intron is not present and the two last exons are fused. ASP5 catalytic residue D431 was mutated to A to create pTub8-ASP5c-D/A-Ty. The Q5 site directed mutagenesis kit (NEB) instructions was followed using primers 4795–4796 with the pTub8-ASP5c-Ty as template. TgGRA24 was amplified from gDNA using primers 4814–4815, and cloned in pTub8TgARO-Myc-Ble [50] between *EcoRI* and *NsiI* sites. This vector was digested with *NotI* and ligated with a PCR product using primer 4943–2642 with pTub5-CAT-Sag1 [52] as template to create the plasmid pTub8-GRA24-Myc-Ble-CAT. To construct the vector pLIC-P_GRA16_-GRA16-3-Myc, the promoter region and the coding sequence were amplified on gDNA using primers 5419–5421 and cloned into the pLIC-3Myc-*dhfr* vector using the LIC cloning method as described [53]. gRNA for CRISPR/Cas9 was generated with primers 4883–4969 on the pSAG1-CAS9gfp-U6gRNA [54] following the Q5 site directed mutagenesis kit (NEB) instructions. The HXGPRT and DHFR cassettes used to generate the CRISPR/Cas9 mediated KOs of ASP5 were amplified by KOD DNA polymerase (Novagen) using primers 5240–5241 and 5142–5143 respectively. PCR products were precipitated in sodium acetate and re-suspended in water prior to transfection.

### Parasite transfection and selection of stable transgenic parasites

*T*. *gondii* tachyzoites were transfected by electroporation as previously described [55]. Selection of transgenic parasites were performed with either mycophenolic acid and xanthine for HXGPRT selection [56], pyrimethamine for DHFR selection [51] or phleomycin for ble selection [21]. All stable expressing strains were cloned by limited dilution in 96-well plates and analyzed for the expression of the transgenes by IFA and for the genomic integration by PCR. The *RHΔku80-DiCre* (abbreviated *RHΔku80)* strain [49] was transfected with 40 μg of the plasmid Ct-ASP-LoxP-YFP-HXGPRT linearized *AvrII*. The resulting strain, *RHΔku80asp5-3Ty*, was transfected with 5’ASP5-pTub8-loxP-KillerRed-Ble linearized *XhoI* to create the *RHΔku80loxPasp5-3Ty* strain. 40 μg of pTub5-Cre [57] was transfected in this strain to obtain *RHΔku80Δasp5* (S1 Fig). To generate *RHΔasp5*, 30 μg of pSAG1-CAS9gfp-U6gASP5 was transfected into *RH* parasites (S2 Fig). Transfected parasites where cloned by GFP+ FACS sorting 48 hr post-transfection. To generate *PRUΔku80Δasp5* and *ME49Δasp5*, 30 μg of pSAG1-CAS9gfp-U6gASP5 and respectively 15 μg of KOD-amplified HXGPRT or DHFR selection cassette flanked by 25 nt homology regions were transfected into *PRUΔku80asp5-3Ty* or *ME49Δasp5* (S2 Fig). Clones were obtained by limiting dilution after appropriate selection. 60 μg of pTub8-ASP5c-Ty, pTub8-ASP5g-Ty, were transfected in *RHΔku80Δasp5* and in *RHΔasp5*. Parasites were passaged for several weeks to allow the Ty positive population to gradually increase. Parasites were cloned and Ty positive clones were selected. 60 μg of pTub8-ASP5c-D/A-Ty was transfected in *RHΔasp5* and HXGPRT-selected. In absence of selection, no Ty positive population was observed even after 6 weeks post-transfection. Transient transfection of GRASP-GFP [58], pTub-GRA19-HA, pTub-GRA19-HA R124A, pTub-GRA20-HA [18] and pLIC-PGRA16-GRA16-3Myc was performed by using 40 μg of each plasmid as previously described [55].

### Plaque assay

A confluent monolayer of HFFs was infected with around 50 freshly egressed parasites for 7 to 8 days before the cells were fixed with PFA/GA. Plaques were visualized by staining with Crystal Violet (0.1%) as previously described [59].

### Intracellular growth assay

*RH*, *RHΔasp5*, *PRUΔku80* and *PRUΔku80Δasp5* were allowed to grow on HFF for 24 hr prior to fixation with PFA/GA. IFA using α-GAP45 antibodies was performed and the number of parasites per vacuole was scored. For each condition, 200 vacuoles were counted. Data are mean value ± s.d. of three independent experiments. Medium depleted in glucose is DMEM 11966 supplemented with up to 6 mM glutamine and 25 μg/ml gentamicin [60].

### Induced egress assay

Freshly egressed tachyzoites were added to a new monolayer of HFF, washed after 30 min and grown for 30 hr. The infected HFF were then incubated for 5 min at 37°C with DMEM containing either 3 μM of the Ca^2+^ ionophore A23187 (from *Streptomyces chartreusensis*, Calbiochem), 50 μM of BIPPO [20] or DMSO as control. Host cells were fixed with PFA/GA, and IFA using α-GAP45 antibodies was performed. 200 vacuoles were counted per strain and per condition, and the number of lysed vacuoles was scored. Data are mean value ± s.d. of three independent experiments.

### Secretion assays

Dense granule secretion assay was performed with freshly egressed parasite washed twice in intracellular buffer (IC; 5 mM NaCl, 142 mM KCl, 2 mM EGTA, 1 mM MgCl_2_, 5.6 mM glucose, 25 mM HEPES-KOH, pH 7.2) containing protease inhibitor cocktail. Parasite constitutive secretion was performed in similar IC buffer. After 1 hr at 37°C, a fraction was collected, which represent the total lysate, and the excreted secreted antigens (ESA) were collected upon sequential centrifugation. Following an initial centrifugation 5 min/4°C/1000 g, supernatants were transferred to a new tube and spun again 5 min/4°C/2000 g. The final supernatant was collected and analyzed by immunoblotting. Processing of the micronemal protein 2 (MIC2) upon secretion of the micronemes was used as control.

### Immunofluorescence assay (IFA)

Antibodies described here were used for IFA and western blot analysis. The mAbs α-Ty tag BB2, α-Myc tag 9E10, α-HA (Covance Inc), α-ROP2-4 T3-4A7 [61], α-MIC2, α-GRA1, α-GRA2, α-GRA3 (J. F. Dubremetz), α-ACT1 [62] as well as the polyclonal Abs α-GAP45 [59], α-CAT [63], α-GRA7 (kindly provided by Prof. D.J.P. Ferguson) α-Hsp70 [64], α-Cpn60 [65] were used. Infected-HFF monolayers on coverslips were fixed with 4% paraformaldehyde (PFA)/0.05% glutaraldehyde (GA) for 10 min or for 30 min for GRAs PV and PVM localization prior to quenching in 0.1M glycine/PBS. Cells were then permeabilized with 0.2% Triton X-100/PBS (PBS/Triton) and blocked in the same buffer supplemented with 2% BSA (PBS/Triton/BSA). Cells were incubated with primary antibodies (Abs) diluted in PBS/Triton X-100/BSA for 1 hr followed by PBS/Triton washes (3 x 5 min). Cells were incubated with secondary Abs (Alexa488- or Alexa594-conjugated goat anti-mouse or goat anti-rabbit IgGs) in PBS/Triton X-100/BSA. Where appropriate, parasite and HFF nuclei staining was performed by incubating cells in DAPI (4’,6-diamidino-2-phenylindole; 50μg/ml in PBS) prior to final washing (3 x 5 min). Coverslips were mounted in Fluoromount G (Southern Biotech) on glass slides and stored at 4°C in the dark. Confocal images were collected with a Zeiss microscope (LSM700, objective apochromat 63x /1.4 oil) at the Bioimaging core facility of the Faculty of Medicine, University of Geneva. Stacks of sections were processed with ImageJ and projected using the maximum projection tool.

### Western blot analyses

Crude extracts of *T*. *gondii* tachyzoites were subjected to SDS-PAGE Western blot analysis carried out using polyacrylamide gels under reducing conditions. Proteins were transferred to hybond ECL nitrocellulose. Primary and secondary antibodies (HRP conjugated, SIGMA) are diluted in PBS, 0.05% Tween20, 5% skimmed milk. Bound antibodies were visualized using the ECL system (Amersham).

### Pulse chase

Metabolic labelling of the tachyzoites was done with 50 mCi [35S]-labeled methionine/cysteine (Hartmann analytic GmbH) per ml for 4 h at 37°C followed by co-IP in RIPA buffer using α-Ty antibodies. A pulse of 7 min was followed by two chases of 15 and 60 min.

### Transmission electron microscopy

Freshly egressed parasites were allowed to invade HFF monolayers for 24 hr prior to fixation. Infected host cells were washed with 0.1M phosphate buffer pH 7.2 and were fixed with 2.5% glutaraldehyde in 0.1M phosphate buffer pH 7.2, post-fixed in osmium tetroxide, dehydrated in ethanol and treated with propylene oxide to embedding in Spurr’s epoxy resin. Thin sections were stained with uranyl actetate and lead citrate prior to examination using a Technai 20 electron microscope (FEI Company). Two independent sample preparation and multiple thin sections for each sample were examined.

### Morphometric analyses

HFF infected with wt or mutant parasites for 24 hr were fixed in 2.5% glutaraldehyde in 0.1 M sodium cacodylate buffer (pH 7.4) for 1 hs at room temperature, and processed as described [66] before examination with a Philips CM120 Electron Microscope (Eindhoven, the Netherlands) under 80 kV. Morphometric analysis to quantify the extent of association of host mitochondria with PV was performed as described [67].

### Chemokine production and quantitative RT-PCR

Total RNA was extracted from infected primary mouse embryonic fibroblasts (pMEFs) using TRIzol reagent. cDNA was synthesized using Verso Reverse transcription (Thermo Fisher Scientific). Real-time PCR was performed using the Go-Taq real-time PCR system (Promega) and the CFX connect real-time PCR system (Biorad). The values were normalized to the amount of actin in each sample. The primer sets used are listed in [28]. Chemokine production was analyzed from three independent experiments.

### Isolation and differentiation of BMDMs

Bone marrow derived macrophages (BMDMs) were obtained by flushing marrow from the hind tibias and femurs of C57Bl/6 mice. The cell suspension was passed through a nylon mesh and cultured in RPMI1640 medium supplemented with 10% FCS, 100 U/ml penicillin, 0.1 mg/ml streptomycin, and 15% L-cell conditioned medium at 37°C degrees in humidified 5% CO_2_. Non-adherent cells were passed the next day to 10-cm bacteriological petri dishes (4.10^6^ cells/dish) and harvested for experiments six days later using a cell scraper.

### IRG recruitment assay

BMDMs were seeded (5.10^5^ per well) in 24 well plates containing coverslips and activated with 10 ng/ml of murine recombinant IFNγ for 24 h at 37°C, 5% CO_2_. The next day, activated cells were infected with freshly egressed and filtered *T*. *gondii* parasites (MOI = 1) for 1 h, then fixed in 4% PFA in PBS for 10 min, semi-permeabilized in 0.002% digitonin in PBS for 7 min at 4°C and blocked in 2% BSA/PBS for 30 min. IFA was carried out using the primary antibodies (Abs) mouse α-Irga6 (kindly provided by Prof. J.C. Howard), goat α-Irgb6 (Santa Cruz), mouse α-GRA1 and mouse α-GRA2. GRA1 and GRA2-containing positive vacuoles were analyzed for the presence of Irgb6 at the vacuole by counting 10 fields at a magnification of 100X. Data was analyzed and images were taken using a confocal laser microscope (FVI1200 IX-83; Olympus) and the software FLUOVIEW (Olympus) and are representative of three independent experiments.

### In vitro cytokine ELISA

BMDMs were seeded (10^5^ per well) in 96-well plates and left to adhere over-night at 37°C, 5% CO_2_. Cells were then infected with freshly egressed and filtered *T*. *gondii* parasites (3.10^5^ parasites per well) and culture supernatants were collected 40 h later and frozen at -20°C degrees. IL-12p40 levels were measured by ELISA according to manufacturer’s instructions in three independent experiments. The assay was also carried out using PECs and RAW264.7 cells.

### RNA isolation

BMDMs were plated to 80% confluence in RPMI with 10% FCS, 100 U/ml penicillin, 0.1 mg/ml streptomycin and infected with the different *T*. *gondii* strains at a MOI of 3. 18–20 hr post-infection, cells were rinsed with cold PBS, detached with trypsin and pelleted. Total RNA from samples in triplicates was extracted using a hybrid RNA extraction protocol with TRIzol (Life Technologies) and QIAGEN RNeasy Mini Kit. Sample pellets were lysed in TRIzol followed by the addition of chloroform to separate the aqueous layer and the organic layer. RNA from the upper aqueous phase was precipitated with 70% ethanol and isolated using the RNeasy column according to the manufacturer's instructions. Isolated RNA was subjected to single read 100 bp on a Illumina HiSeq 2500 at the Genomics platform at the University of Geneva, iGE3 (the Institute of Genetics and Genomics). The RNA samples were multiplexed across 3 sequencing lanes of the flow cell.

### RNA Sequencing and data analysis

RNA Sequencing was performed on the Illumina HiSeq 2500 at the iGE3 genomics plateform of the University of Geneva (http://www.ige3.unige.ch/genomics-platform.php). The adapter sequences from the raw reads obtained from the RNA-Seq were trimmed using FASTX-Toolkit (http://hannonlab.cshl.edu/fastx_toolkit/) (phred<20). The resulting reads, after quality control, were aligned to the latest mouse reference genome (GRCm38) using TopHat/Bowtie2 aligner and HTSeq-count was used to get the read counts of the genes [68–71]. Differential expression analysis was carried out using edgeR, a Bioconductor package in R (http://www.R-project.org). Normalized expression values from the count data were obtained from the normalization factors calculated by the TMM (trimmed mean of the M values) method. The heatmaps for the genes of interest were also generated in R using heatmap.2 in the gplots package. KEGG (Kyoto Encyclopedia of Genes and Genomes) pathway analysis was performed using KOBAS 2.0 (KEGG Orthology Based Annotation System) [72, 73]. All the computations were performed at University of Geneva on the Baobab cluster.

### Isolation and differentiation of BM-derived dendritic cells (DC)

BM-DCs were obtained as previously described [74]. Briefly, DCs were generated by culturing BM cells for 7–10 days in the presence of 20 ng/ml GMCSF in RPMI supplemented with 10% heat-incativated FCS, 50 mM 2-mercaptoethanol, 100 mM sodium pyruvate and 100 μM penicillin/streptomycin at 37°C in humidified 5% CO_2_.

### DC motility assay

DC (3–5.10^4^) were challenged with freshly egressed tachyzoites at a MOI of 3, treated with LPS (100 ng/ml) or maintained in complete medium (non-infected). Cells were settled on gelatin-coated glass slides for 6–8 hr at 37°C. The cells were imaged every min for 45–60 min (Zeiss Cell Observer.Z1). Motility patterns were compiled using ImageJ (image stabilizer software and manual tracking plugins).

### DC transmigration assay

DCs were plated at a density of 1.10^6^ cells/well and incubated with freshly egressed *T*. *gondii* tachyzoïtes (MOI 3) for 4 hr at 37°C and 5% CO_2_. DCs were then transferred into transwell filters (8 μm pore size; Corning) and incubated for 16 hr at 37°C and 5% CO_2_. Migrated DC were quantified in a hematocytometer.

### Bradyzoite differentiation assay

In vitro tachyzoite to bradyzoite conversion was induced by exposing parasite cultures to pH 8.2 as described previously [75]. Briefly, 5.10^4^ tachyzoites were allowed to infect HFF grown on glass coverslips inside 24-well plates. 24 hr post infection, bradyzoite differentiation was induced by replacing normal media with RPMI 1640 buffered with 50 mM HEPES to pH 8.2 and supplemented with 3% fetal bovine serum. Parasites were allowed to grow at 37°C in absence of CO_2_ for 4 days and alkaline media was changed daily. After 4 days of conversion, infected HFF were fixed with 3.7% formaldehyde, permeabilized with 0.5% Triton X-100 in phosphate buffered saline (PBS) for 20 min. After 1 hr incubation with 10% foetal calf serum (FCS) as blocking agent, the cells were stained for 1 hr with DBA conjugated with Alexa 594 (used at 10 μg/mL; Vector) and with α-BAG1 mAb (kindly provided by Prof. V.B. Carruthers) followed by Alexa Fluor 594 goat anti-rabbit IgG antibody 200 vacuoles were counted from 20 fields for each experiment to determine the positive/negative rate of DBA and BAG1 staining.

### Mice experiments

Mice were infected by intraperitoneal injection. The health of the mice was monitored daily until they presented severe symptoms of acute toxoplasmosis (bristled hair and complete prostration with incapacity to drink or eat) and were sacrificed on that day.

### Ethics statement

All animal experiments were conducted with the authorization Number (1026/3604/2, GE30/l3) according to the guidelines and regulations issues by the Swiss Federal Veterinary Office. No human samples were used in these experiments. Human foreskin fibroblasts (HFF) were obtained from ATCC.

## Supporting Information

S1 Fig (A) *RHΔku80asp5-3Ty* was investigated by pulse chase to assess to origin of the short-ASP5 band. A pulse of 7 min followed by a 15 min and 60 min chase did not revealed any conversion from the upper to the lower band suggesting that both products are made independently. (B) Complementation of *RHΔasp5* with the catalytic dead ASP5-D/A does not rescue the impairment in the lytic cycle due to ASP5 deletion. Deletion of ASP5 in the type II parasite ME49 resulted in a marked impairment of the lytic cycle. Plaque assay were fixed after 9 days. Mean area of 10 plaques ± s.d.is depicted. (C) Intracellular growth with type I and type II parasites was assessed after 24 hr in different glucose-depleted media. Parasites lacking ASP5 were not impacted in their ability to replicate intracellularly. The branched-chain alpha-keto acid dehydrogenase knock-out [60], previously demonstrated to be more susceptible in glucose depleted media, was used as control. Data are mean value ± s.d. of three experiments. (D) During natural egress, a significant fraction of the parasites remains attached together, forming sphere-like structures (black arrowhead) while wt egressed tachyzoites were individualized (white arrowhead). (E) Deletion of ASP5 in *PRUΔku80* does not affect the morphology of the micronemes (α-MIC2), the rhoptries (α-ROP2-4), the mitochondrion (α-HSP70) and the apicoplast (α-Cpn60). Deletion of ASP5 resulted in a significant impairment of the lytic cycle, as assessed by plaque formation after 7 days, in both type I and II parasites.
(TIF)

S2 Fig (A) Schematic representation of the strategy used to generate the *RHΔku80asp5-3Ty*, *RHΔku80loxPasp5-3Ty*, and *RHΔku80Δasp5* strains. Plasmids Ct-ASP-LoxP-YFP-HXGPRT and 5’ASP5-pTub8-loxP-KillerRed-Ble were transfected sequentially in the *RHΔku80DiCre* strain (abbreviated *RHΔku80*, in this manuscript) to obtain the first two strains. Unfortunately, Cre dimerization by rapamycin was no more responsive in this strain. Alternatively, we transfected the pTub5-Cre [57] plasmid and FACS sorted (Moflo-Astrios, Beckman Coulter) the resulting YFP+/DsRed- parasites. Parasites were cloned in 96-wells plate to obtain *RHΔku80Δasp5*. (B) PCR analyses on the different strains generated in (A).
(TIF)

S3 Fig (A) CRISPR/Cas9 gRNA design used to generate the *RHΔasp5*, *PRUΔku80Δasp5* and *ME49Δasp5*. PAM, protospacer adjacent motif (red); CRISPR, clustered regularly interspaced short palindromic repeats; Cas9, CRISPR associated protein 9. (B) Sequencing of the gDNA region of *RHΔasp5* targeted with the CRISPR/Cas9 strategy revealed an insertion mutation resulting with a premature stop codon (*). (C) *PRUΔku80asp5-3Ty* was first generated using the DHFR selection cassette. In a second step, the HXGPRT selection cassette was amplified by KOD DNA polymerase with 25 nt ASP5 homology arms in both 5’ and 3’. The CRISPR/Cas9 strategy was used to enhance integration of the cassette at the targeted locus. Correct 5’ integration was demonstrated by PCR. Unexpectedly, the promoter region of the HXGPRT recombined in the DHFR promoter (both cassette have the same promoter). This resulted in the inversion of the ASP5 sequence as demonstrated by PCR analyses. Clone #1 was used for the analyses presented in the manuscript. (D) The same strategy than in (C) was used to disrupt ASP5 except that the DHFR cassette was used instead. PCR analyses showed correct integration in both 5’ and 3’ and the absence of the endogenous locus. Clone #1 was used for the analyses presented in the manuscript.
(TIF)

S4 Fig Electron micrographs of the membranous nanotubular network (MNN, black arrows) in type I parasites. When not completely missing, flawed MNN is observed in parasites lacking ASP5 (black asterisk). Very short tubules/small vesicles fill the vacuolar space of *RHΔasp5* parasites. Scale bars represent 1 μm.
(TIF)

S5 Fig Serum collected from surviving mice to *ME49Δasp5* infection were tested by immunoblot for seroconversion on HFF cells only or extracellular *RH* tachyzoites material. (TIF)

S6 Fig (A) Heatmap of selected, statistically overrepresented, pathways (p value < 0.05) identified by KEGG enrichment analysis of differentially expressed genes (>2fold, p-value < 0.05). Genes listed in S1 Table, Sheet 4 and 5 were analyzed and comparisons were made between BMDMs infected with type I and type II wt or *Δasp5* strains and non-infected BMDMs. Differentially expressed genes were identified using edgeR and TMM normalized expression values (TMM normalized RNA-Seq read counts) were used to building the heatmap by the gplots package in R. The normalized gene expression values (averaged over the replicates) were log2 transformed and scaled and clustered row-wise. (B) Venn diagram showing the number of differentially expressed genes common between the different comparisons.
(TIF)

S1 Table Results of differential expression analysis between wt and *Δasp5* infected BMDMs and results of KEGG pathway enrichment analysis (p<0.05) on the differentially expressed genes. A threshold of fold change >2 and p-value < 0.05 was used for subsequent analysis.
(XLSX)

S2 Table Primers used in this study. (DOCX)

S1 Movie (S1) During egress from infected host cell, parasites lacking ASP5 were often found trapped in sphere-like structures (white arrows). This phenotype is not fully understood, but parasites seem to be unable to rapidly escape from the double membrane system, the PVM and the host plasma membrane, which surround them during egress. S1 was recorded during 40s. N: host-cell nucleus.
(AVI)
