# Supplementary figures and images for: Fundamental Roles of the Golgi-Associated Toxoplasma Aspartyl Protease, ASP5, at the Host-Parasite Interface
Source: PLoS Pathog. 2015 Oct 16;11(10):e1005211. doi: 10.1371/journal.ppat.1005211 (PMC4608785; doi:10.1371/journal.ppat.1005211)

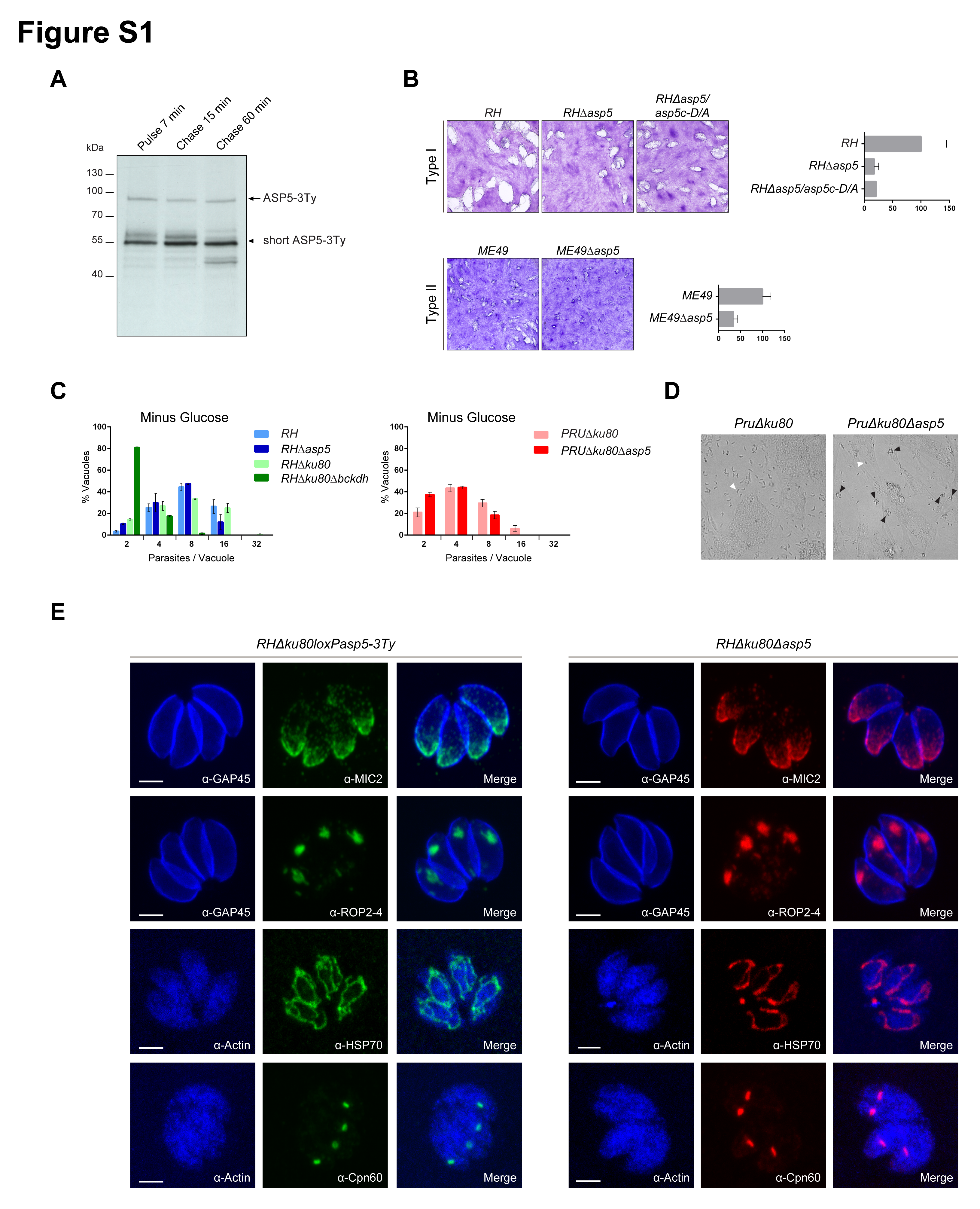

Supplement: S1 Fig — A pulse of 7 min followed by a 15 min and 60 min chase did not revealed any conversion from the upper to the lower band suggesting that both products are made independently. (B) Complementation of RHΔasp5 with the catalytic dead ASP5-D/A does not rescue the impairment in the lytic cycle due to ASP5 deletion. Deletion of ASP5 in the type II parasite ME49 resulted in a marked impairment of the lytic cycle. Plaque assay were fixed after 9 days. Mean area of 10 plaques ± s.d.is depicted. (C) Intracellular growth with type I and type II parasites was assessed after 24 hr in different glucose-depleted media. Parasites lacking ASP5 were not impacted in their ability to replicate intracellularly. The branched-chain alpha-keto acid dehydrogenase knock-out [60], previously demonstrated to be more susceptible in glucose depleted media, was used as control. Data are mean value ± s.d. of three experiments. (D) During natural egress, a significant fraction of the parasites remains attached together, forming sphere-like structures (black arrowhead) while wt egressed tachyzoites were individualized (white arrowhead). (E) Deletion of ASP5 in PRUΔku80 does not affect the morphology of the micronemes (α-MIC2), the rhoptries (α-ROP2-4), the mitochondrion (α-HSP70) and the apicoplast (α-Cpn60). Deletion of ASP5 resulted in a significant impairment of the lytic cycle, as assessed by plaque formation after 7 days, in both type I and II parasites. (TIF) [file ppat.1005211.s001.tif]

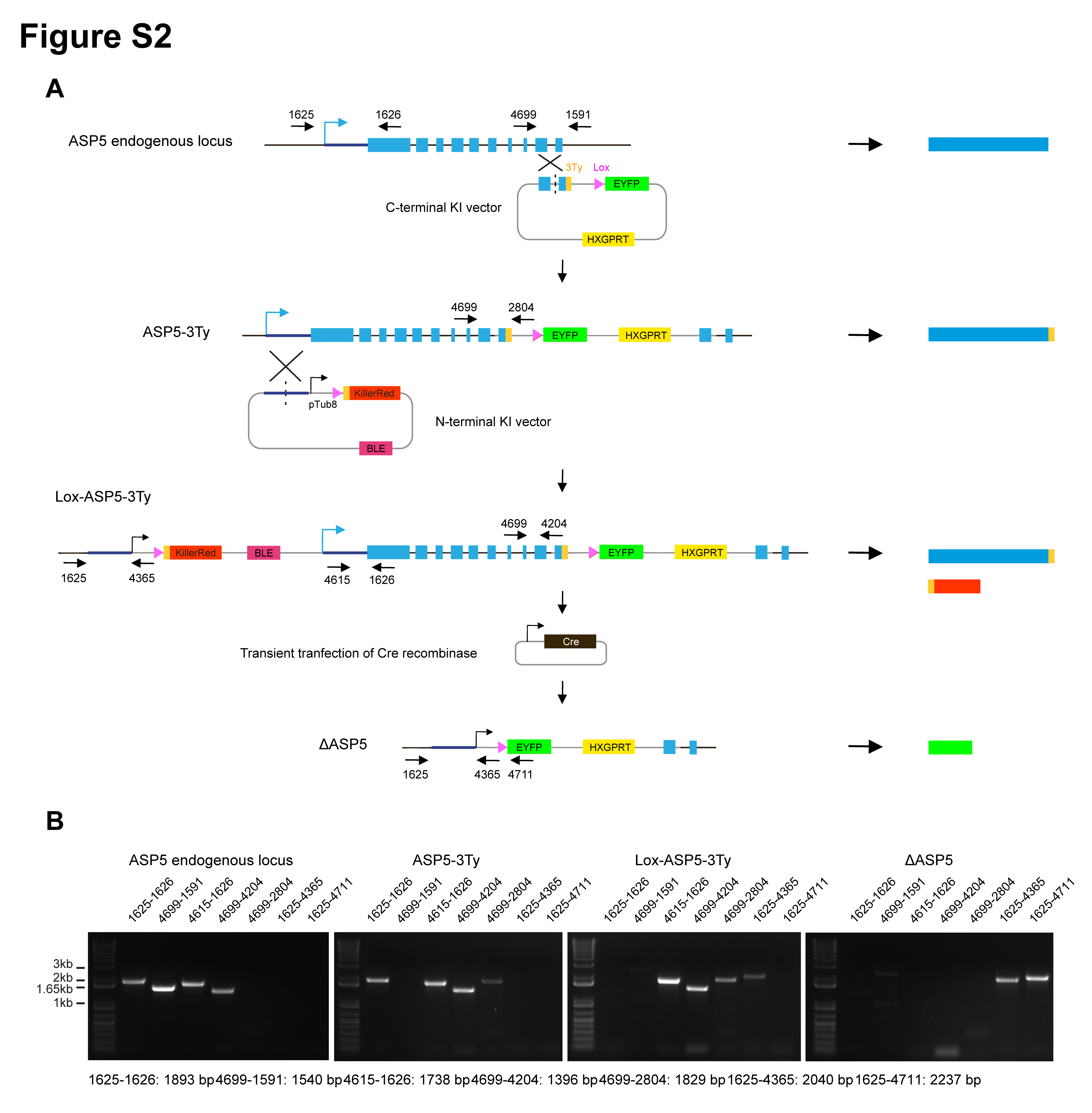

Supplement: S2 Fig — Plasmids Ct-ASP-LoxP-YFP-HXGPRT and 5’ASP5-pTub8-loxP-KillerRed-Ble were transfected sequentially in the RHΔku80DiCre strain (abbreviated RHΔku80, in this manuscript) to obtain the first two strains. Unfortunately, Cre dimerization by rapamycin was no more responsive in this strain. Alternatively, we transfected the pTub5-Cre [57] plasmid and FACS sorted (Moflo-Astrios, Beckman Coulter) the resulting YFP+/DsRed- parasites. Parasites were cloned in 96-wells plate to obtain RHΔku80Δasp5. (B) PCR analyses on the different strains generated in (A). (TIF) [file ppat.1005211.s002.tif]

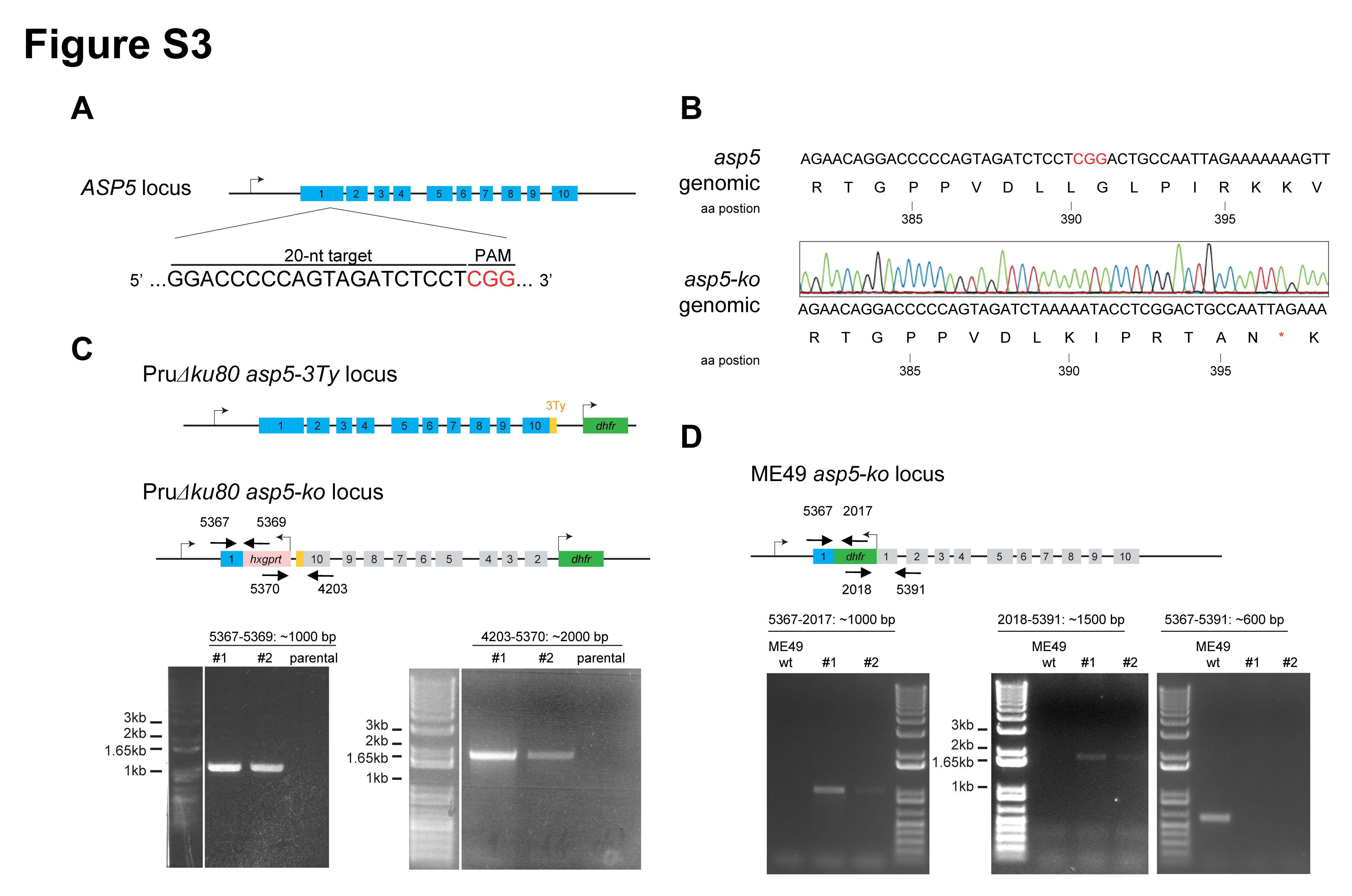

Supplement: S3 Fig — PAM, protospacer adjacent motif (red); CRISPR, clustered regularly interspaced short palindromic repeats; Cas9, CRISPR associated protein 9. (B) Sequencing of the gDNA region of RHΔasp5 targeted with the CRISPR/Cas9 strategy revealed an insertion mutation resulting with a premature stop codon (*). (C) PRUΔku80asp5-3Ty was first generated using the DHFR selection cassette. In a second step, the HXGPRT selection cassette was amplified by KOD DNA polymerase with 25 nt ASP5 homology arms in both 5’ and 3’. The CRISPR/Cas9 strategy was used to enhance integration of the cassette at the targeted locus. Correct 5’ integration was demonstrated by PCR. Unexpectedly, the promoter region of the HXGPRT recombined in the DHFR promoter (both cassette have the same promoter). This resulted in the inversion of the ASP5 sequence as demonstrated by PCR analyses. Clone #1 was used for the analyses presented in the manuscript. (D) The same strategy than in (C) was used to disrupt ASP5 except that the DHFR cassette was used instead. PCR analyses showed correct integration in both 5’ and 3’ and the absence of the endogenous locus. Clone #1 was used for the analyses presented in the manuscript. (TIF) [file ppat.1005211.s003.tif]

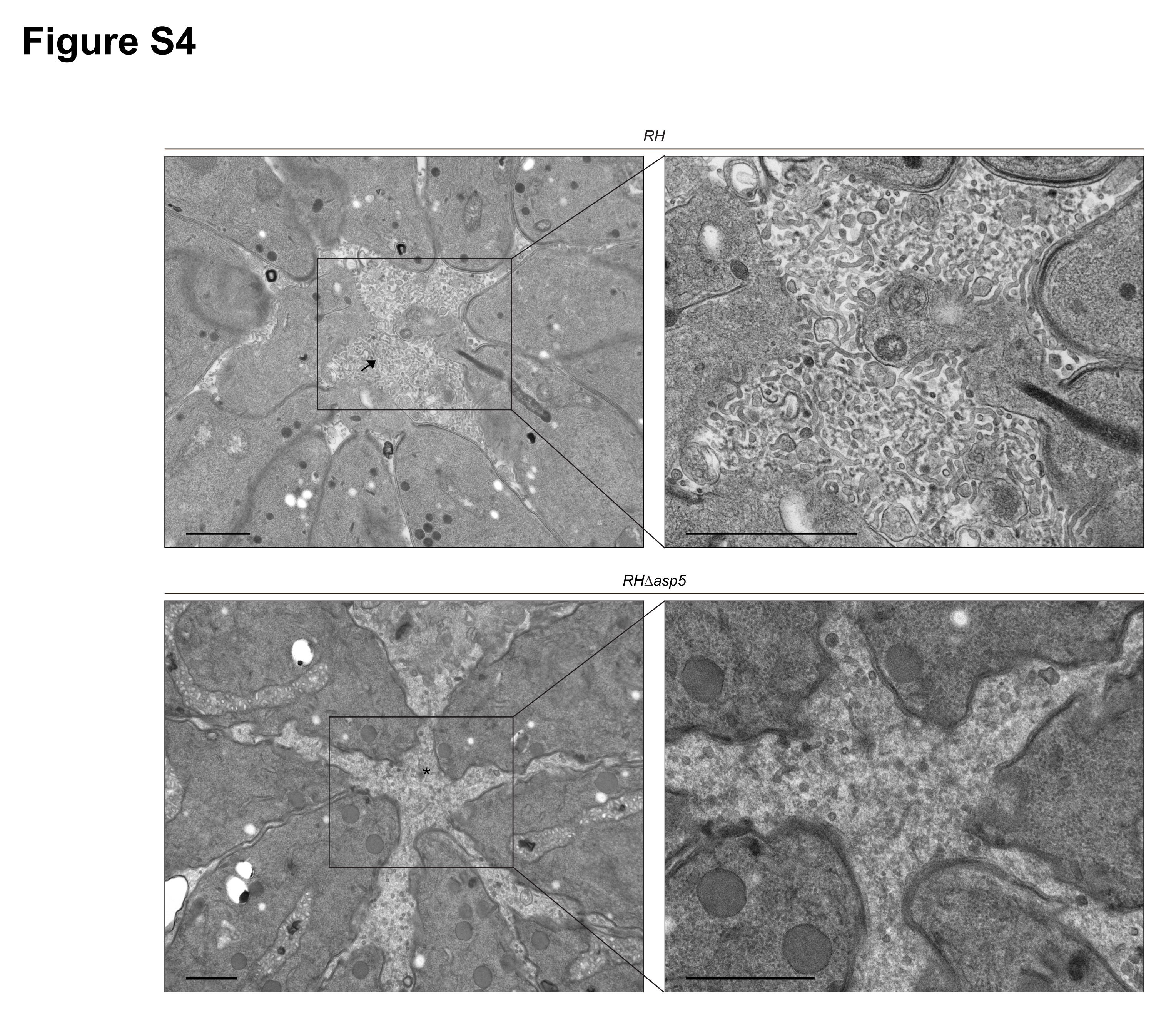

Supplement: S4 Fig — When not completely missing, flawed MNN is observed in parasites lacking ASP5 (black asterisk). Very short tubules/small vesicles fill the vacuolar space of RHΔasp5 parasites. Scale bars represent 1 μm. (TIF) [file ppat.1005211.s004.tif]

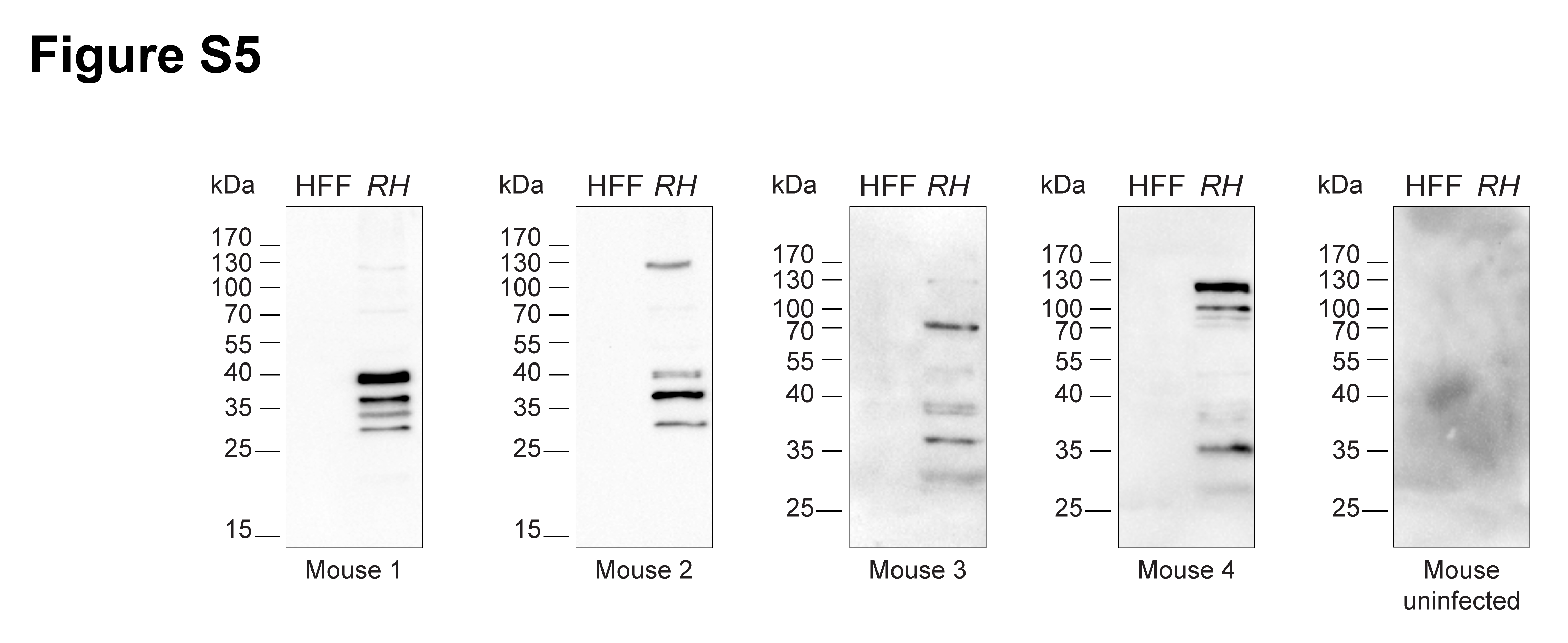

Supplement: S5 Fig — (TIF) [file ppat.1005211.s005.tif]

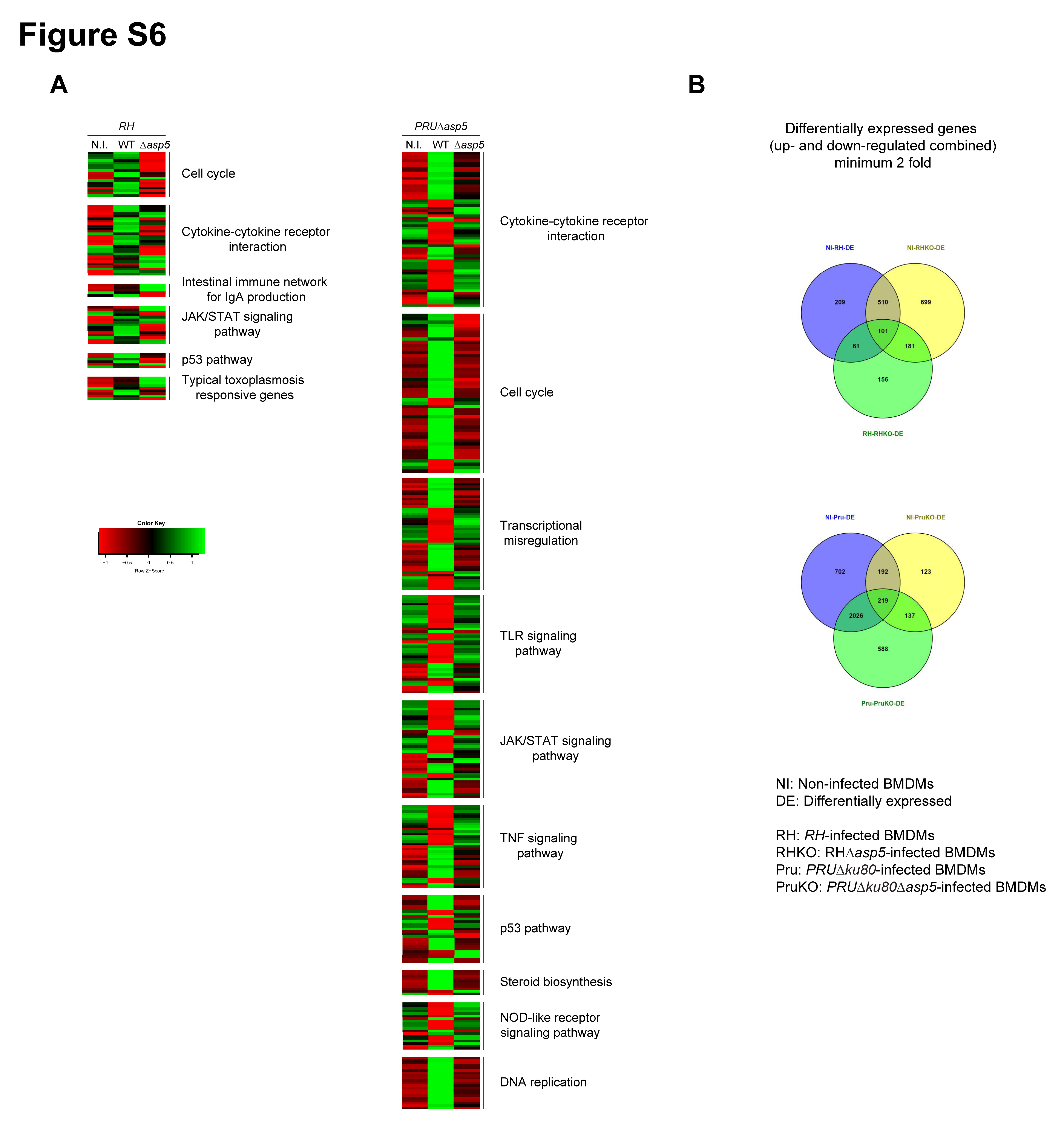

Supplement: S6 Fig — Genes listed in S1 Table, Sheet 4 and 5 were analyzed and comparisons were made between BMDMs infected with type I and type II wt or Δasp5 strains and non-infected BMDMs. Differentially expressed genes were identified using edgeR and TMM normalized expression values (TMM normalized RNA-Seq read counts) were used to building the heatmap by the gplots package in R. The normalized gene expression values (averaged over the replicates) were log2 transformed and scaled and clustered row-wise. (B) Venn diagram showing the number of differentially expressed genes common between the different comparisons. (TIF) [file ppat.1005211.s006.tif]
