# Supplementary material for: Fundamental Roles of the Golgi-Associated Toxoplasma Aspartyl Protease, ASP5, at the Host-Parasite Interface
Source: PLoS Pathog. 2015 Oct 16;11(10):e1005211. doi: 10.1371/journal.ppat.1005211 (PMC4608785; doi:10.1371/journal.ppat.1005211)
Supplement: S2 Table — (DOCX) [file ppat.1005211.s008.docx]

**Table S2.** Primers used in this study.

| **Primer name** | **Nr** | **Enzyme** | **Sequence (5'-3')** | **Resulting plasmid** |
| --- | --- | --- | --- | --- |
| TgGRA24.1 | 4814 | EcoRI | CGGGAATTCCAGTGATGCTCCAGATGGCACGATATAC | pT8-GRA24-Myc-BLE |
| TgGRA24.2 | 4815 | NsiI | CGGATGCATAATTACCCTTAGTGGGTGGTTTAACGATGC | pT8-GRA24-Myc-BLE |
| TgGRA16.1 | 5419 | - | TACTTCCAATCCAATTTAGCCATTTTGAAACGTGAGCGAGTAG | pLIC-P_GRA16_-GRA16-3Myc |
| TgGRA16.2 | 5421 | - | TCCTCCACTTCCAATTTTAGCCATCTGATCATTTTTCCGCTTCGC | pLIC-P_GRA16_-GRA16-3Myc |
| TgAsp5.17 | 4203 | ApaI | GCCGGGCCCCCGACTGTGTCTTGTTTGCGGCTG | Ct-ASP5-3Ty-HX |
| TgAsp5.18 | 4204 | NsiI | GCCATGCATACTCTCCGCCAAGAGGCAGATCC | Ct-ASP5-3Ty-HX |
| TgAsp5.43 | 4615 | ApaI | AGCGGGCCCTCCATTCCAGCTCTCGGAGG | 5’ASP5-pTub8-loxP-KillerRed-Ble |
| TgAsp5.44 | 4616 | ApaI | ATTGGGCCCATGGCTAAAAGGTGGAGCACTC | 5’ASP5-pTub8-loxP-KillerRed-Ble |
| TgAsp5.7 | 1624 | MfeI | CGCCAATTGCCTTTTTCGACAAAATGGAGGCAGGCGCGATGG | pTub8-ASP5g-Ty |
| TgAsp5.6 | 1592 | NsiI | GGCATGCATTCTCTCCGCCAAGAGGCAGATC | pTub8-ASP5g-Ty |
| TgAsp5-DA.1 | 4795 | - | CGTGCGTCTGTCATTCTTGCCACCGGAAGTTCTCTCC | pTub8-ASP5c-D/A-Ty |
| TgAsp5-DA.2 | 4796 | - | GGAGAGAACTTCCGGTGGCAAGAATGACAGACGCACG | pTub8-ASP5c-D/A-Ty |
| CAT | 4943 | NotI/SacII | TCTGCGGCCGCCCGCGGCCCCCCCTCGAGGTC | pTub8-GRA24-Myc-Ble-CAT |
| Sag1-3’UTR | 2642 | NotI | ACCGCGGTGGCGGCCGCTC | pTub8-GRA24-Myc-Ble-CAT |
| TgAsp5-HX.1 | 5240 | - | AGACATTTCCGGCAGCGTCAGGAGTCGTCCGCGGTGTCACTGTAGCCTGC | Flanked HXGPRT cassette |
| TgAsp5-HX.2 | 5241 | - | ACCGGCGCATGGGAAGGCGAGGAGAGAACTCCGATCTTGCTGCTGTTCGC | Flanked HXGPRT cassette |
| TgAsp5-DHFR.1 | 5142 | - | AGACATTTCCGGCAGCGTCAGGAGTCGTCCGCGGCCGCTCTAGAACTAG | Flanked DHFR cassette |
| TgAsp5-DHFR.2 | 5143 | - | ACCGGCGCATGGGAAGGCGAGGAGAGAACTGCGGAAGATCCGATCTTGC | Flanked DHFR cassette |
| gRNA.1 | 4883 | - | AACTTGACATCCCCATTTAC | CRISPR/Cas9 |
| TgAsp5.gRNA | 4969 | - | GGACCCCCAGTAGATCTCCTGTTTTAGAGCTAGAAATAGC | CRISPR/Cas9 |
| TgAsp5.8 | 1625 | ApaI | GCGGGCCCCCTTCGCGTTTTCTCCGCGAC | Primer for screen |
| TgAsp5.9 | 1626 | - | CCTTGCCGCTTCTTCTCTCGC | Primer for screen |
| TgAsp5.50 | 4699 | - | GGTCTGCCATTATCCCCTCAACG | Primer for screen |
| TgAsp5.5 | 1591 | - | CGAAAGACACTGGCGCACGC | Primer for screen |
| TgAsp5.43 | 4615 | ApaI | AGCGGGCCCTCCATTCCAGCTCTCGGAGG | Primer for screen |
| TgAsp5.8 | 1625 | ApaI | GCGGGCCCCCTTCGCGTTTTCTCCGCGAC | Primer for screen |
| TgAsp5.9 | 1626 | - | CCTTGCCGCTTCTTCTCTCGC | Primer for screen |
| TgAsp5.18 | 4204 | NsiI | GCCATGCATACTCTCCGCCAAGAGGCAGATCC | Primer for screen |
| TgAsp5.51 | 2804 | XbaI | CCGTCTAGACTCGGGGGGGCAAGAATTGTG | Primer for screen |
| pTub-Rev | 4365 | StuI | CGGAGGCCTAGAAAAAATGCCAACGAGTAGTTTTCC | Primer for screen |
| YFP | 4711 | - | CTTGCCGGTGGTGCAGATGAACTTC | Primer for screen |
| TgAsp5.52 | 5367 | - | GGCTCTGCTCCTCGCGACTGTC | Primer for screen |
| HXGPRT.1 | 5369 | - | AGATGTTCCGCGACTTCGACCAC | Primer for screen |
| HXGPRT.2 | 5370 | - | GCCGTAGTCTTCAATGGGTTTGG | Primer for screen |
| TgDHFR.1 | 2017 | - | GTCACTTGTTGTGCCAGTTCTAC | Primer for screen |
| TgDHFR.2 | 2018 | - | CTTGGGGGTCATCGCGACGACCAGAC | Primer for screen |
| TgAsp5.53 | 5391 | - | CACGGTATAATTCTTAACATGGTCGGA | Primer for screen |
